# Optimal exercise dose-response improves health-related quality of life in cancer survivors: a systematic review and Bayesian network meta-analysis of RCTs

**DOI:** 10.3389/fonc.2024.1510578

**Published:** 2024-12-16

**Authors:** Zhiyu Xiong, Yuan Yuan, Yong Yang, Bopeng Qiu, Ying Bai, Tao Wang, Junyu Wang, Lin Zhang, Yawen Li

**Affiliations:** ^1^ The School of Physical Education and Health, East China Jiaotong University, Nanchang, China; ^2^ The School of Physical Education, Kunsan National University, Kunsan-si, Jeollabuk-do, Republic of Korea; ^3^ Laboratory of Kinesiology and Rehabilitation, School of Physical Education and Sport, Chaohu University, Hefei, China; ^4^ School of Strength and Conditioning Training, Beijing Sport University, Beijing, China; ^5^ College of Physical Education and Health, Southwest University of Science and Technology, Mianyang, China; ^6^ The School of Exercise and Health, Shanghai University of Sport, Shanghai, China; ^7^ Department of Rehabilitation, West China Hospital Sichuan University Jintang Hospital, Chengdu, China; ^8^ The School of Electrical & Automation Engineering, East China Jiaotong University, Nanchang, China

**Keywords:** cancer survivor, exercise, health-related quality of life, dose-response, Bayesian network meta-analysis

## Abstract

**Objective:**

Cancer survivors often face significant health-related quality of life (HRQoL) challenges. Although exercise has been proven to improve HRQoL in cancer survivors, the optimal dose and intensity of exercise for this population has not been fully determined. Adherence to exercise may vary based on exercise intensity, affecting results. This study explored the dose-response relationship of different exercise types and intensities to better understand their impact on HRQoL in cancer survivors.

**Methods:**

We searched five databases—PubMed, Embase, the Cochrane Library, Web of Science, and Scopus—from their inception until 1 December 2023. Data analysis was performed using R software with the MBNMA and RJAGS packages. Due to combining data from different scales, effect sizes were reported as standardized mean differences (SMD) with 95% credible intervals (95% CrI). The risk of bias was assessed independently by three reviewers using the RoB2 tool.

**Results:**

A total of 48 studies involving 3050 cancer survivors. Across all exercise types, the most beneficial exercise dose was identified to be 850 metabolic equivalents of task (METs)-min/week (SMD: 0.753, 95%Crl: 0.463 to 1.096), with diminishing returns observed beyond 1,100 METs-min/week. Among the various types of exercises, mixed training (MT) emerged as the optimal choice, demonstrating its efficacy at 970 METs-min/week (SMD: 0.883, 95% Crl: 0.455 to 1.345). Aerobic exercise (AE) at a dose of 430 METs-min/week (SMD: 0.681, 95% Crl: 0.206 to 1.099) and resistance training (RT) at 450 METs-min/week (SMD: 0.695, 95% Crl: 0.227 to 1.203) also showed significant benefits. Additionally, mind-body exercises, such as tai chi, qigong, or yoga, exhibited optimal effects at a dose of 390 METs-min/week (SMD: 0.672, 95% Crl: 0.259 to 1.087).

**Conclusion:**

Our study sheds light on the intricate relationship between exercise interventions and health-related quality of life in cancer survivors, as elucidated through a systematic review and Bayesian network meta-analysis. The identified optimal exercise dose of 850 METs-min/week resulted in a significant improvement in health-related quality of life, underscoring the importance of regular exercise in cancer survivorship. MT emerged as the most effective modality, closely followed by RT, AE, and MBE.

**Systematic review registration:**

https://www.crd.york.ac.uk/prospero/display_record.php?RecordID=493328, identifier CRD42024493328.

## 1 Introduction

Cancer is a group of diseases characterized by abnormal cell growth. It is one of the deadliest diseases worldwide, claiming several million lives annually (1, 2). According to research statistics from 185 countries for 36 kinds of cancers in 2020, there were approximately 19.3 million cases of cancer and nearly 10 million cancer-related deaths worldwide, and the incidence rates of cancer in both men and women were two to three times higher in developing countries than in developed countries (3). Because of the strong influence of the demographic shift towards aging on the varying trends in cancer incidence in different regions, the incidence of all cancers is projected to double by 2070 compared with 2020 (4). In addition, the global economic cost of cancer from 2020 to 2050 is estimated to be US$25.2 trillion which is equivalent to an annual tax on the global gross domestic product of 0.55% (5). Although medical treatment has significantly improved the survival rate of cancer patients in recent years, the symptoms that arise after treatment can seriously impact the quality of life of these patients. Therefore, it is crucial from both clinical and public health perspectives to eliminate the negative effects of cancer in all aspects.

Exercise is increasingly recognized as a non-pharmacological intervention with profound implications for improving the quality of life across a spectrum of chronic diseases, including metabolic syndrome-related disorders, cardiovascular and pulmonary diseases, muscular and joint disorders, and cancer (6, 7). In the case of exercise in oncology, a multitude of preclinical studies have consistently demonstrated the anti-tumor effects of different types of exercise across various cancer models (8, 9). Furthermore, adding to the body of evidence, a review published in the Journal of Natural Metabolism systematically explored the pathogenic factors of cancer and proposed a reprogramming strategy involving the immune and metabolic networks stimulated by exercise. This not only reinforces the connection between exercise and the suppression of tumorigenesis but also underscores the logical coherence underlying the role of exercise in combating cancer (10).

Health-related quality of life (HRQoL) serves as the primary endpoint for evaluating the well-being of cancer survivors, encompassing the impact of the disease and its treatments on physical, psychological, and social dimensions (11). Consequently, a growing number of researchers are increasingly directing their attention toward understanding and enhancing HRQoL among cancer survivors. For example, in a previous study investigating the efficacy of exercise in improving overall HRQoL among adult cancer survivors post-treatment, it was concluded that exercise indeed had a positive impact, particularly evident in addressing cancer-specific concerns (12). Furthermore, a recent network meta-analysis encompassing 93 studies with 7,435 cancer patients further reinforced these findings. It demonstrated that a combination of aerobic and resistance exercises significantly enhances HRQoL in both the during and post-treatment phases, highlighting its clinical significance (13).

There is evidence of the effectiveness of exercise in improving the HRQoL of cancer patients, and studies have confirmed the effectiveness of specific exercise forms. However, there are still research gaps regarding the optimal exercise dose. To improve precision exercise interventions for cancer survivors’ HRQoL, we used metabolic equivalents of task (METs) for different exercise doses (14) and explored the optimal dose-response relationship between exercise and HRQoL.

Our study is a systematic review and used a new dose-response Bayesian model network meta-analysis (MBNMA) method (15). The aim was to quantify the available evidence from randomized controlled trials that exercise improves HRQoL in cancer survivors. We used the new method to not only explore the optimal mode of exercise to improve the quality of life of cancer survivors but also identify the optimal exercise dose that improves cancer survivors’ HRQoL.

## 2 Methods

This systematic review and network meta-analysis was registered with the International Prospective Register of Systematic Reviews (PROSPERO: CRD 42024493328) and this NMA followed the Preferred Reporting Items for Systematic Review and Meta-analysis protocols statement extension (PRISMA-NMA) checklist (16).

### 2.1 Data sources and search strategy

A systematic search for studies was carried out in the PubMed, Embase, the Cochrane Library, Web of Science, and Scopus databases from their inception to 1 December 2023. The search strategy was structured according to the Peer Review of Electronic Search Strategies 2015 guidelines (17). We provide a complete list of search times, search entries, and search results in the **Supplementary Material** (**Supplementary Table 1**). Two investigators (ZX and YY) independently screened the title/abstract and full text of the articles, with any disagreements resolved by discussion or adjudication by a third author (YL).

### 2.2 Study selection

According to the principles of the “PICOS” inclusion criteria (18), the included studies comprised the following criteria: (a) Population: cancer survivors over 18 years of age; (b) Intervention: Any form of exercise; (c) Outcome: Measure the health-related quality of life in cancer survivors [ex: Functional Assessment of Cancer Therapy-General (Fact-General) (19); 36-Item Short Form Health Survey (SF-36) (20); European Organization for Research and Treatment of Cancer Quality of Life Questionnaire-Core 30 (EORTC QLQ-C30) (21); World Health Organization Quality of Life-BREF (WHOQOL-BREF) (22)]; (d) Study design: Must be randomized controlled trial (RCT); (e): Comparison: Controls could be daily care, physical health education, or other forms of exercise.

The exclusion criteria were as follows: (a) studies on children with cancer and cancer patients undergoing treatment; (b) intervention studies combining exercise with cognitive therapy, physiotherapy, massaging, diet, or medication; (c) studies that excluded trials with no control group; (d) endpoints that reported cancer-specific scales and excluded non-cancer survivors’ HRQoL scales; and (e) individuals with major depression, cognitive impairment or other diseases that may affect exercise ability and quality of life.

### 2.3 Data extraction and definition

Two authors independently extracted data from the included studies (ZX and YY), and all authors resolved disagreements by consensus. First, the following data from each included study were extracted: first author and year of publication, sample (sample size), region (study country), age (patient age), cancer type, intervention characteristics (intervention duration/time, type of exercise), and HRQoL-related outcome indicators.

Second, data were extracted as means and standard deviations (SD) of the HRQoL-related outcome indicators before and after the intervention, based on the Cochrane Handbook for Systematic Evaluation of Intervention (23). To meet the data analysis requirements of the dose-response network meta-analysis package in the R program, we also converted the standard errors (SE). SE=SD/SQRT (Sample size/N) (24). The relevant data are provided in **Supplementary File 3**. When it was not possible to retrieve the minimum required data from a published report for the dose-response meta-analysis, we contacted the authors and invited them to provide additional data.

### 2.4 Data setting and management

First, we coded the interventions in a first level of categorization to classify all the interventions as equivalent to analyze the dose-response effect of overall exercise on the HRQoL of cancer survivors. Second, to analyze the optimal dose and the different forms of exercise, a second level of classification coding was performed on the interventions: Aerobic exercise (AE) (treadmill training, dance; stationary bike, etc.), mind-body exercise (MBE) (yoga; taiji; qigong);, mixed training (MT) (combined aerobic exercise and resistance exercise), resistance training (RT), and control (Placebo).

Previous studies have demonstrated the good efficacy of metabolic equivalents of task (METs) in evaluating exercise intensity (25, 26). Thus, in our study, the term “dose” referred to energy expenditure expressed in METs-min/week. We used the method validated by Ainsworth et al. (14) to calculate the different doses associated with each intervention in our study and to calculate the product of the duration of the exercise intervention, the number of sessions, and the metabolic equivalent of the task (METs-min/week). In order to better promote the connectivity of the network, we used an approximate value of 250, 500, 750, 1000, or 1500 METs-min/week for the exercise dose, which was adopted from previous studies (27).

### 2.5 Data synthesis

We used the Bayesian-Model of network meta-analysis package (MBNMA) (28), and “rjags” package (29) in R version 4.0.3. (R Foundation for Statistical Computing, Vienna, Austria). We conducted the network meta-analysis to compare the overall relative effectiveness and different dose responses of the interventions under investigation. We checked the connectivity of the motion pattern grid for different motion doses based on the dose approximation (30) and we obtained agreement between the two models by comparing them with an uncorrelated mean-effects model (UME) (**Supplementary File 4.1**) (31). Finally, we assessed transitivity using a node-splitting approach (**Supplementary File 4.2**) (32). There were no parametric results that indicated a violation of the above key assumptions.

Regarding the dose-response models, firstly, to identify the observed effect of the overall exercise dose and different exercise doses on HRQoL, we plotted a confirmation of the non-linear relationship between overall exercise dose and cancer survivors’ HRQoL improvement (**Supplementary File 5 Figures 1, 2**).

To summarize the dose-response relationship between exercise and cancer survivors’ HRQoL, we compared dose-response models (Emax, Exponential, Restricted cubic spline, non-parametric, and linear models) regarding fit indices, including the deviance information criterion (DIC), between-study standard deviation, the number of parameters in the models, and the residual values. Ultimately, we opted for the random effect of restricted cubic splines to evaluate the non-linear dose-response association, as detailed in **Supplementary File 5 Table 5.1**.

In order to visualize our study data and model-fitting results, deviance plots were used to confirm the robustness of our model selection (**Supplementary File 5 Figures 3, 4**) (33). We employed a Markov Chain Monte Carlo (MCMC) model, utilizing three chains with 20,000 iterations each (the first 10,000 discarded) and a thinning factor of 10. Through this model, we leveraged Beta coefficients derived from restricted cubic spline curves to estimate both the overall and varying effects of exercise doses on HRQoL, identifying the exercise dose associated with the optimal effect. Additionally, we placed nodes at the 10th, 50th, and 90th percentiles of the exercise dose to effectively visualize the results of our model fitting process (15). Our study considered different outcome measures. Therefore, we chose the standardized mean difference (SMD) (34) as the effect size for our study. We used a 95% credible interval (95% CrI) to provide a range of values within which we are confident the true effect size lies (35). The code to reproduce the results presented in this paper can be requested by contacting the first author’s e-mail address.

### 2.6 Risk of bias

It was essential to identify and address the fundamental assumptions of heterogeneity, inconsistency, and consistency. Therefore, we first used the tau-squared (τ²) test and I² statistic to evaluate the statistical heterogeneity between the studies. Moreover, we assessed global inconsistency statistically using the design-by-treatment interaction test. We separated indirect from direct evidence using the SIDE (Separating Indirect from Direct Evidence) test using the “netmeta” package. Additionally, we compared adjusted funnel plots to assess the risk of publication bias under specific circumstances. Egger’s test indicated bias when p<0.05.

Additionally, the risk of bias was assessed according to the second version of the Cochrane risk-of-bias tool for randomized trials (RoB 2) (36). Three reviewers (BQ, YB, and TW) assessed the study. The assessment items included randomized sequence generation, bias due to deviation from the intended intervention, incomplete data, bias in measurements, and selective bias in reporting results. And conducted a risk assessment of our research based on the following items:

- Random sequence generation. This domain assesses whether the process of randomizing participants was adequately described and whether the random sequence generation method was likely to produce comparable groups. Studies were classified as having low, unclear, or high risk of bias based on the transparency and appropriateness of the randomization process.
- Bias due to deviations from intended interventions. This domain assesses whether participants and personnel were blinded to the interventions and whether deviations from the intended interventions could have affected the study results. Risks were categorized based on the degree of blinding and the handling of deviations from the intervention protocol.
- Incomplete data. This domain considers whether the study had complete outcome data or if there were substantial follow-up losses that could affect the reliability of the results. Risks were assessed based on how missing data were handled and the impact on the study conclusions.
- Bias in outcome measurement. This domain assesses whether the methods used to measure the outcomes were appropriate and whether there was a risk of bias in the measurement process. Studies were rated based on whether the outcome measurements were objectively consistent or if there was a risk of bias due to subjective assessment methods.
- Selective reporting bias. This domain assesses whether there was evidence of the selective reporting of outcomes, such as reporting only some outcomes and omitting others. Studies were rated based on whether all prespecified outcomes were reported and whether there was any evidence of selective reporting.

Disagreements between reviewers were resolved by the third author (JW), who was responsible for re-examining controversial assessments and discussing with other reviewers to reach a consensus, ensuring the accuracy and consistency of the assessment results.

### 2.7 Sensitivity analysis

To assess the potential impact of high risk of bias on our findings, we performed sensitivity analyses in which we excluded studies assessed to be at high risk of bias and reanalyzed the dose-response data.

## 3 Results

### 3.1 Basic characteristics of the included studies

**Figure 1:**
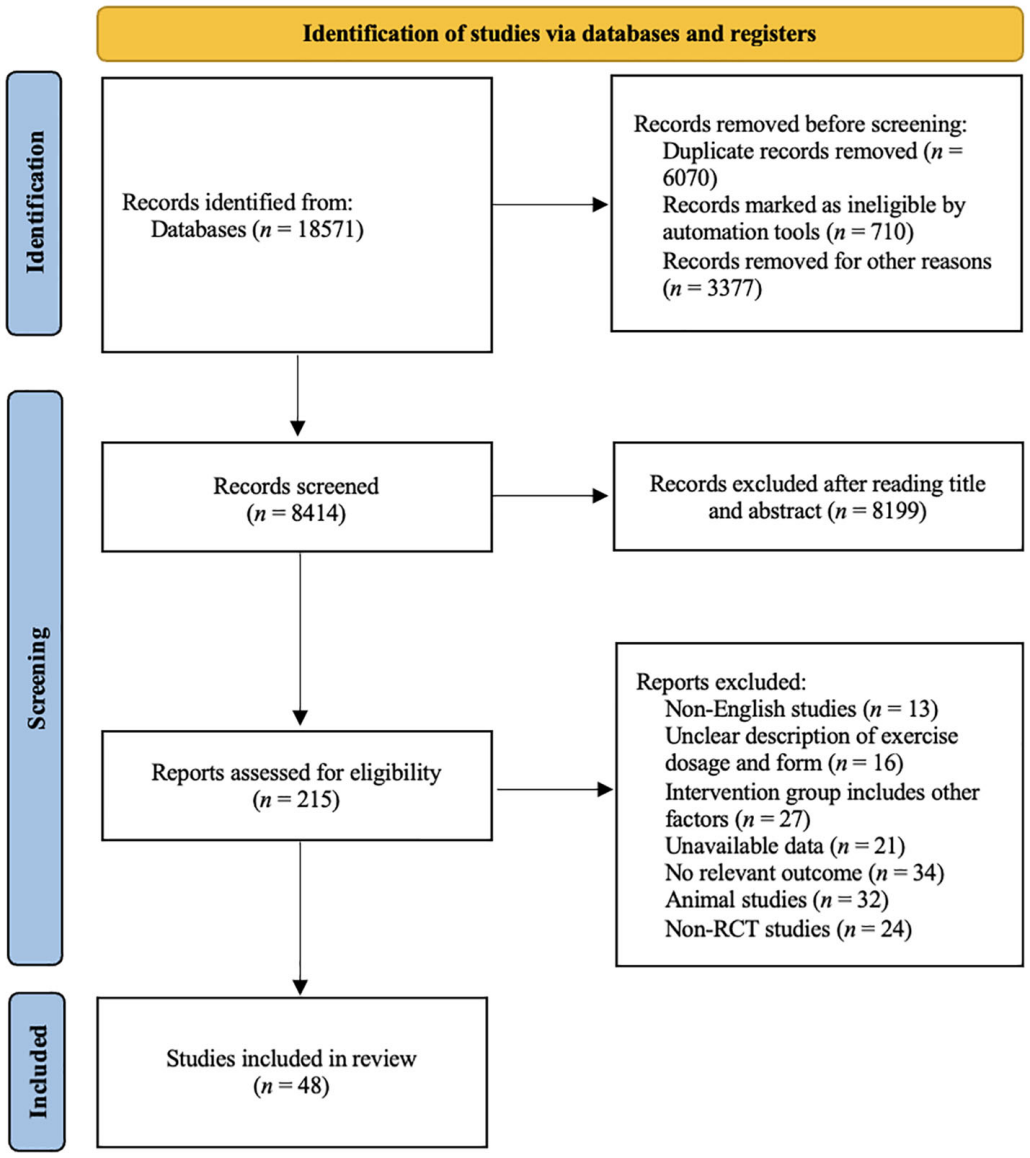
PRISMA flow diagram of the search process for studies. RCT, randomized controlled trials.

A total of 18,571 study records were found. After removing duplicates and literature that did not fit the study by title, abstract, etc., we considered 215 full-text articles that were then screened for eligibility. **Figure 1** shows a flow diagram of the study selection process. Finally, 48 studies involving 3050 participants were included in our network meta-analysis. The patients included in the studies had different types of cancer and were aged between 44 to 72 years old. The basic characteristics of the included population are given in **Table 1**.

**Table 1:** Studies on interventions and outcomes in cancer patients.

Study	Sample	Age	Cancer	Intervention	Intervention details	Outcome	METs-min	Intervention duration/ time	Region
Kerry et al., 2003	24	59 ± 5	Breast	AE	Upright cycle ergometers (Bicycle 25min)1-3 (15min)/13-15 (35min)	FACT-G	6.8	15weeks/3times	Canada
	28	58 ± 6	Breast	CON	CON	FACT-G	0	15weeks/3times	Canada
Courneya et al., 2004	62	59.2 ± 10.73	Colorectal	AE	AE (Swimming、Cycling)25min	FACT-G	4	16weeks/3times	Canada
	31	61.13 ± 9.93	Colorectal	CON	CON	FACT-G	0	16weeks/3times	Canada
Tetsuya et al., 2006	39	53.3 ± 8.7	Breast	RT	RT (30min)	QoL CARES-SF	3.5	13weeks/2times	USA
	40	52.8 ± 7.6	Breast	CON	CON	QoL CARES-SF	0	13weeks/2times	USA
Nicole et al., 2006	18	51.18 ± 10.33	Breast	MBE	Yoga 75min	Global health status (EORTC QLQ-C30)	2.8	7weeks/4times	Canada
	18	50.46 ± 8.2	Breast	CON	CON	Global health status (EORTC QLQ-C30)	0	7weeks/4times	Canada
Kavita et al., 2014	53	52.38 ± 1.35	Breast	MBE	Yoga 60min	SF-36	2.3	6weeks/3times	USA
	56	51.14 ± 1.32	Breast	CON	Stretch 60min	SF-36	2.3	6weeks/3times	USA
Helen et al., 2008	29	55.2 ± 8.4	Breast	MT	AE 20min (Cycle/ergometer (Treadmill)+RT(30min)	FACT-General	3.5	12weeks/3times	Australia
	29	55.1 ± 8	Breast	CON	CON	FACT-General	0	12weeks/3times	Australia
Seung et al., 2010	13	47.5 ± 5.1	Breast	RT	RT (40min)	Global health status (EORTC QLQ-C30)	5	8weeks/1time	Korea
	18	47 ± 9.2	Breast	CON	CON	Global health status (EORTC QLQ-C30)	0	8weeks/1time	Korea
Lisa et al., 2012	9	54.33 ± 3.55	Breast	MBE	Taichi (60min)	SF-36	3	12weeks/3times	USA
	10	52.7 ± 2.11	Breast	CON	CON	SF-36	0	12weeks/3times	USA
Alyson et al., 2012	32	60.6 ± 7.7	Breast	MBE	Yoga(75min)	FACT-General	2.3	24weeks/5times	USA
	31	58.2 ± 8.8	Breast	CON	CON	FACT-General	0	24weeks/5times	USA
Siedentopf et al., 2013	33	55.82 ± 10.72	Breast	MBE	Yoga (60min)	Global health status (EORTC QLQ-C30)	4	5weeks/2times	Germany
	21	58.41 ± 9.91	Breast	CON	CON	Global health status (EORTC QLQ-C30)	0	5weeks/2times	Germany
Bernardine et al., 2013	20	59.5 ± 11.2	Colorectal	AE	AE (Walking、Cycle) 30min	SF-36	4.8	12weeks/5times	USA
	26	55.6 ± 8.24	Colorectal	CON	CON	SF-36	0	12weeks/5times	USA
Broderick et al., 2013	23	52.3 ± 8.3	Mixed	AE	AE(Brisk walking、Treadmill、Bicycle) 45min	FACT-General	3.5	8weeks/2times	Ireland
	20	51.2 ± 10.3	Mixed	CON	CON	FACT-General	0	8weeks/2times	Ireland
Streckmann et al., 2014	26	44(20-67)	Lymphoma	MT	AE/Sensorimotor training/ST 60min	EORTC-QLQ-C-30(QoL)	3.9	36weeks/2times	Germany
	25	48(19-73)	Lymphoma	CON	CON	EORTC-QLQ-C-30(QoL)	0	36weeks/2times	Germany
Ardiana et al., 2014	30	53 ± 11	Breast	AE	AE(Treadmill、Stationary bicycles)35min	FACT-General	4.8	10weeks/3times	Kosovo
	32	51 ± 11	Breast	CON	CON	FACT-General	0	10weeks/3times	Kosovo
Wiebke et al., 2014	11	58.7 ± 12	Gastrointestinal	RT	RT 45min	Global health status (EORTC QLQ-C30)	3.5	12weeks/2times	Germany
	10	51.6 ± 13.6	Gastrointestinal	AE	AE (Bicycle ergometer 45min)	Global health status (EORTC QLQ-C30)	4.3	12weeks/2times	Germany
Chang et al., 2014	32	62 ± 12.15	Lung	AE	AE (Brisk walking) 6 minutes	WHOQ OL-BREF(QoL)	4.3	12weeks/7times	China
	33	58.39 ± 13.39	Lung	CON	CON	WHO QOL-BREF(QoL)	0	12weeks/7times	China
Barbara et al., 2014	32	64 ± 10	Lung	MT	AE Stationary bicycles (20min) RT (15min)	SF-36	4.15	10weeks/1times	Denmark
	35	65 ± 9	Lung	CON	CON	SF-36	0	10weeks/1times	Denmark
Noémie et al., 2015	87	49.7 ± 8.2	Breast	MT	AE/Sensorimotor training/RT 30 min	Global health status (EORTC QLQ-C30)	4	36weeks/2times	Netherlands
	77	49.5 ± 7.9	Breast	CON	CON	Global health status (EORTC QLQ-C30)	0	36weeks/2times	Netherlands
Amerigo et al., 2016	17	64 ± 10	Endometrial	MT	Dance 25min and RT 20min	FACT-G	4	12weeks/2times	USA
	12	65 ± 5	Endometrial	CON	CON	FACT-G	0	12weeks/2times	USA
Luca et al., 2016	10	50.2 ± 9.7	Breast	MT	AE (cycle-ergometer) and RT (70min-40min 30min)	FACT-G	4	24weeks/2times	Italy
	10	46.0 ± 2.8	Breast	CON	CON	FACT-G	0	24weeks/2times	Italy
Vanderbyl et al., 2017	11	66.1 ± 11.7	Breast	MBE	Qigong 45min	FACT-G	3	6weeks/2times	Canada
	13	63.7 ± 7.7	Breast	MT	mix (AE/RT 2-4 Mets 45min)	FACT-G	4	6weeks/2times	Canada
Maria et al., 2017	13	56.7 ± 8.6	Mixed	AE	Dance 45min	SF-36	3	12weeks/1times	USA
	16	59 ± 10	Mixed	CON	CON	SF-36	0	12weeks/1times	USA
Maximilian et al., 2018	30	54.2 ± 7.8	Breast	MBE	Taichi(90min)	Global health status (EORTC QLQ-C30)	3	24weeks/2times	Germany
	21	51.5 ± 8.4	Breast	CON	CON	Global health status (EORT QLQ-C30)	0	24weeks/2times	Germany
Anne et al., 2018	50	53 ± 8.9	Breast	MT	AE(Cycle-ergometer) and RT 60 minutes	Global health status (EORTC QLQ-C30)	4	12weeks/3times	Belgium
	51	53.7 ± 9.8	Breast	CON	CON	Global health status (EORTC QLQ-C30)	0	12weeks/3times	Belgium
Christina et al., 2018	50	53.5 ± 10.4	Breast	MT	(AE and RT)—50 min	FACT-G	4	16weeks/3times	USA
	50	NA	Breast	CON	CON	FACT-G	0	16weeks/3times	USA
Colleen et al., 2018	38	51.42 ± 12.6	Mixed	MT	AE and RT 90 min	SF-36	4	12weeks/2times	Canada
	39	55.03 ± 11.76	Mixed	CON	CON	SF-36	0	12weeks/2times	Canada
Brigitta et al., 2019	21	67.6 ± 4.6	Prostate	MT	Exergaming (60min)	Global health status (EORTC QLQ-C30)	7.2	12weeks/3times	Denmark
	20	69.8 ± 4.4	Prostate	CON	CON	Global health status (EORTC QLQ-C30)	0	12weeks/3times	Denmark
Nilofar et al., 2019	12	51.6 ± 10.46	Breast	MBE	Yoga	Global health status (EORTC QLQ-C30)	2.8	8weeks/3times	Iran
	15	51.8 ± 11.4	Breast	CON	CON	Global health status (EORTC QLQ-C30)	0	8weeks/3times	Iran
Sara et al., 2019	74	52.7 ± 10.3	Breast	RT	RT 60min	Global health status (EORTC QLQ-C30)	6	16weeks/2times	Sweden
	72	51.8 ± 11.4	Breast	AE	AE (interval aerobic exercise)	Global health status (EORTC QLQ-C30)	4	16weeks/2times	Sweden
	60	52.6 ± 10.2	Breast	CON	CON	Global health status (EORTC QLQ-C30)	0	16weeks/2times	Sweden
Jesper et al., 2019	19	57.8 ± 10.4	Colon	AE	Interval walking	FACT-G	4.8	150min/week	Denmark
	20	60.3 ± 8.9	Colon	CON	CON	FACT-G	0	150min/week	Denmark
Cešeiko et al., 2019	27	48.2 ± 6.7	Breast	RT	20minRT	Global health status (EORTC QLQ-C30)	5	12weeks/2times	Latvia
	28	49.0 ± 8.0	Breast	CON	CON	Global health status (EORTC QLQ-C30)	0	12weeks/2times	Latvia
Morten et al., 2020	110	65.2 ± 8.2	Lung	MT	AE and RT and strength 90min	FACT-G	4	12weeks/2times	Denmark
	108	63.5 ± 8.7	Lung	CON	CON	FACT-G	0	12weeks/2times	Denmark
Hong et al., 2020	94	55.4 ± 11.6	Gastrointestinal	RT	RT60min	Global health status (EORTC QLQ-C30)	3.5	12weeks/2times	China
	96	52.3 ± 12.4	Gastrointestinal	CON	CON	Global health status (EORTC QLQ-C30)	0	12weeks/2times	China
Elise et al., 2020	4	68 ± 6.4	Lung and colorectal	AE	Nordic Walking 30 minutes	SF-36	4.8	8weeks/3times	Canada
	4	67 ± 6	Lung and colorectal	CON	CON	SF-36	0	8weeks/3times	Canada
Abbas et al., 2020	35	69.4 ± 5.77	Prostate	MT	60min/week AE (Low-Moderate walking) RT and Flexibility	Global health status (EORTC QLQ-C30)	4	12weeks/2times	Iran
	36	70.39 ± 5.45	Prostate	CON	CON	Global health status (EORTC QLQ-C30)	0	12weeks/2times	Iran
Lisa et al., 2021	12	64 ± 14	Rectal	AE	AE (30min cycle ergometer)	Global health status (EORTC QLQ-C30)	4	9weeks/3times	UK
	11	57 ± 10	Rectal	CON	CON	Global health status (EORTC QLQ-C30)	0	9weeks/3times	UK
Lin et al., 2021	20	52.1 ± 15.7	Head and Neck	MT	AE 30 Min Treadmill /RT 15 min Blastid band	Global health status (EORTC QLQ-C30)	3	8weeks/3times	China
	20	54.3 ± 9.9	Head and Neck	CON	CON	Global health status (EORTC QLQ-C30)	0	8weeks/3times	China
Roxanne et al., 2021	67	58 ± 9.8	Breast	MT	AE 30Min Treadmill /RT30min	Global health status (EORTC QLQ-C30)	4	12weeks/2times	Netherlands
	114	58.3 ± 9.5	Breast	CON	CON	Global health status (EORTC QLQ-C30)	0	12weeks/2times	Netherlands
Sibel et al., 2021	15	51.40 ± 10.6	Breast	MBE	Yoga (60min)	Global health status (EORTC QLQ-C30)	2.5	10weeks/2times	Turkey
	16	58.3 ± 9.5	Breast	CON	CON	Global health status (EORTC QLQ-C30)	0	10weeks/2times	Turkey
Denise et al., 2021	10	61.00 ± 12.12	Lung	AE	AE (60min)	Global health status (EORTC QLQ-C30)	4.8	12weeks/2times	China
	9	61.11 ± 7.01	Lung	MBE	Taichi 60min	Global health status (EORTC QLQ-C30)	3	12weeks/2times	China
	11	58.36 ± 9.32	Lung	CON	CON	Global health status (EORTC QLQ-C30)	0	12weeks/2times	China
Marta et al., 2022	34	72.1	Mixed	MT	AE (15minstationary bicycling) RT 35min.stretch10min	Global health status (EORTC QLQ-C30)	3.7	12weeks/2times	Denmark
	29	71.5	Mixed	CON	CON	Global health status (EORTC QLQ-C30)	0	12 weeks/2times	Denmark
Dejan et al., 2022	12	52.5	Mixed	AE	AE (braked cycle ergometers)60min	Global health status (EORTC QLQ-C30)	3.5	12 weeks/2times	Germany
	12	58	Mix cancer	CON	CON	Global health status (EORTC QLQ-C30)	0	12 weeks/2times	Germany
Dharam et al., 2022	14	55–60.5	Prostate	MBE	Yoga 60min	FACT-G	2.5	6weeks/2times	USA
	15	59–61	Prostate	CON	CON	FACT-G	0	6weeks/2times	USA
Pedro et al., 2023	20	66.4 ± 7.2	Lung	MT	30min (walking) /2 session (RT)	Global health status (EORTC QLQ-C30)	4	4week/2times	Portugal
	20	68.7 ± 10.3	Lung	CON	CON	Global health status (EORTC QLQ-C30)	0	4week/2times	Portugal
Joachim et al., 2016	13	53 ± 8	Mix	AE	HIIT-5min (warming up) +eight-1min (intense walking) +2min (slow walking)	EORTC QLQ-C30 Global health status	4.3	3weeks/3times	Germany
	13	54 ± 9	Mix	AE	CON	NA	3.8	NA	Germany
Holyan et al., 2017	30	53.17 ± 7.66	Breast	MBE	Pilates +RT (45min)	EORTC QLQ-BR23	3	8weeks/3times	USA
	30	54.03 ± 12.57	Breast	RT	CON	NA	4	NA	USA
Alyson et al., 2007	45	55.11 ± 10.07	Breast	MBE	Yoga 90min	FACT-G	2.8	12weeks/more than one class	USA
	26	54.23 ± 9.81	Breast	CON	CON	NA	0	NA	USA
Jonna et al., 2015	17	58.1 ± 10.3	Colon	MT	Warming up (10 min), Aerobic and muscle strength training (40 min), and a cooling down (10 min)	Global health status (EORTC QLQ-C30)	4	18weeks/more than one class	Netherlands
	16	58.1 ± 9.6	Colon	CON	CON	Global health status (EORTC QLQ-C30)	0	NA	Netherlands

### 3.2 Network connectivity

**Figure 2:**
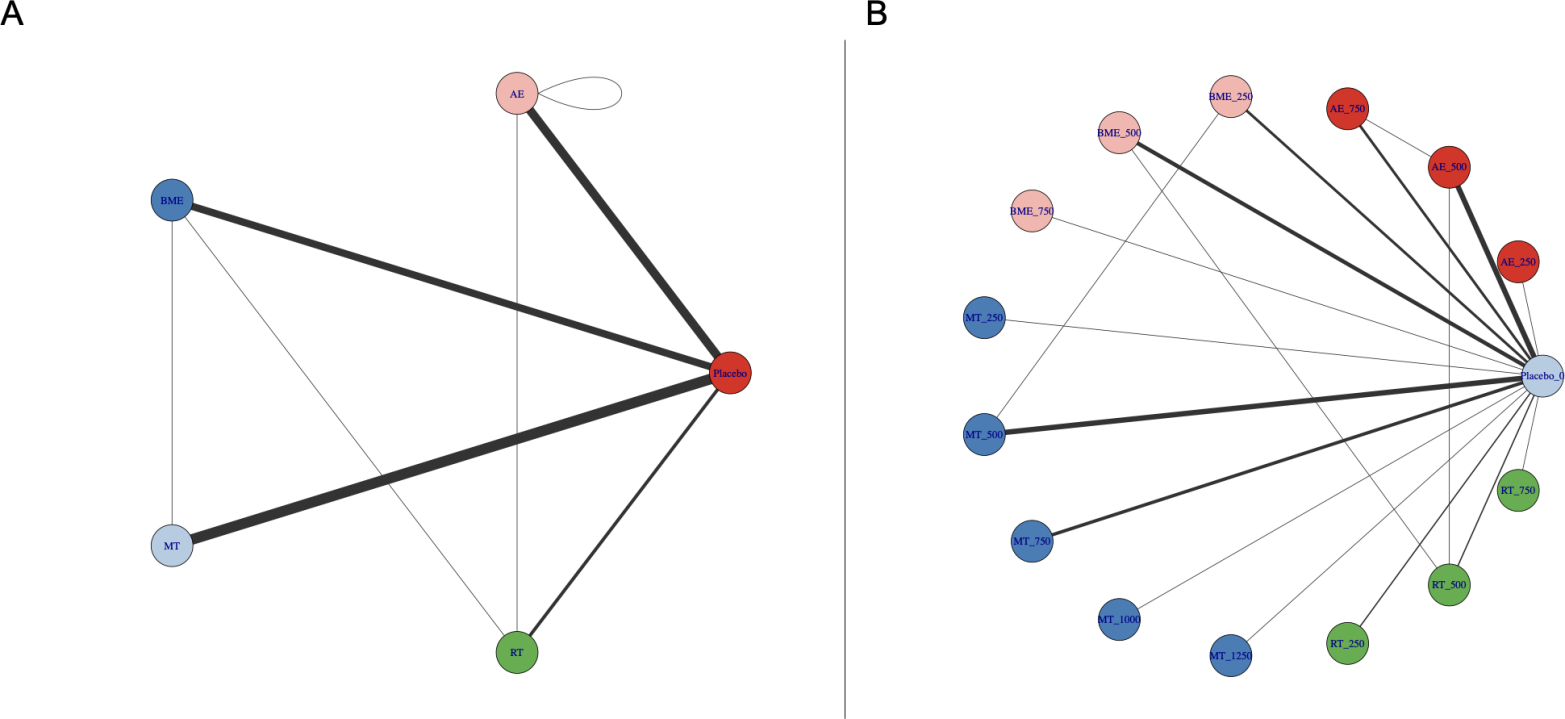
Treatment-level **(A)** and agent-level network plots **(B)** for specific interventions. AE, aerobic exercise; RT, resistance training; MBE, mind-body exercise; MT, mixed training (aerobic + resistance training); CON, control group. Exercise dose corresponds to different exercise types (METs-min/week).

Whether connectivity was met is the basis of NMA. A lack of connectivity can lead to low statistical power and misleading results when a direct comparison is not possible (32). The analysis confirmed that there was no connectivity deficit in the two networks, ensuring accurate results (**Figure 2**).

### 3.3 Dose-response relationship

**Figure 3:**
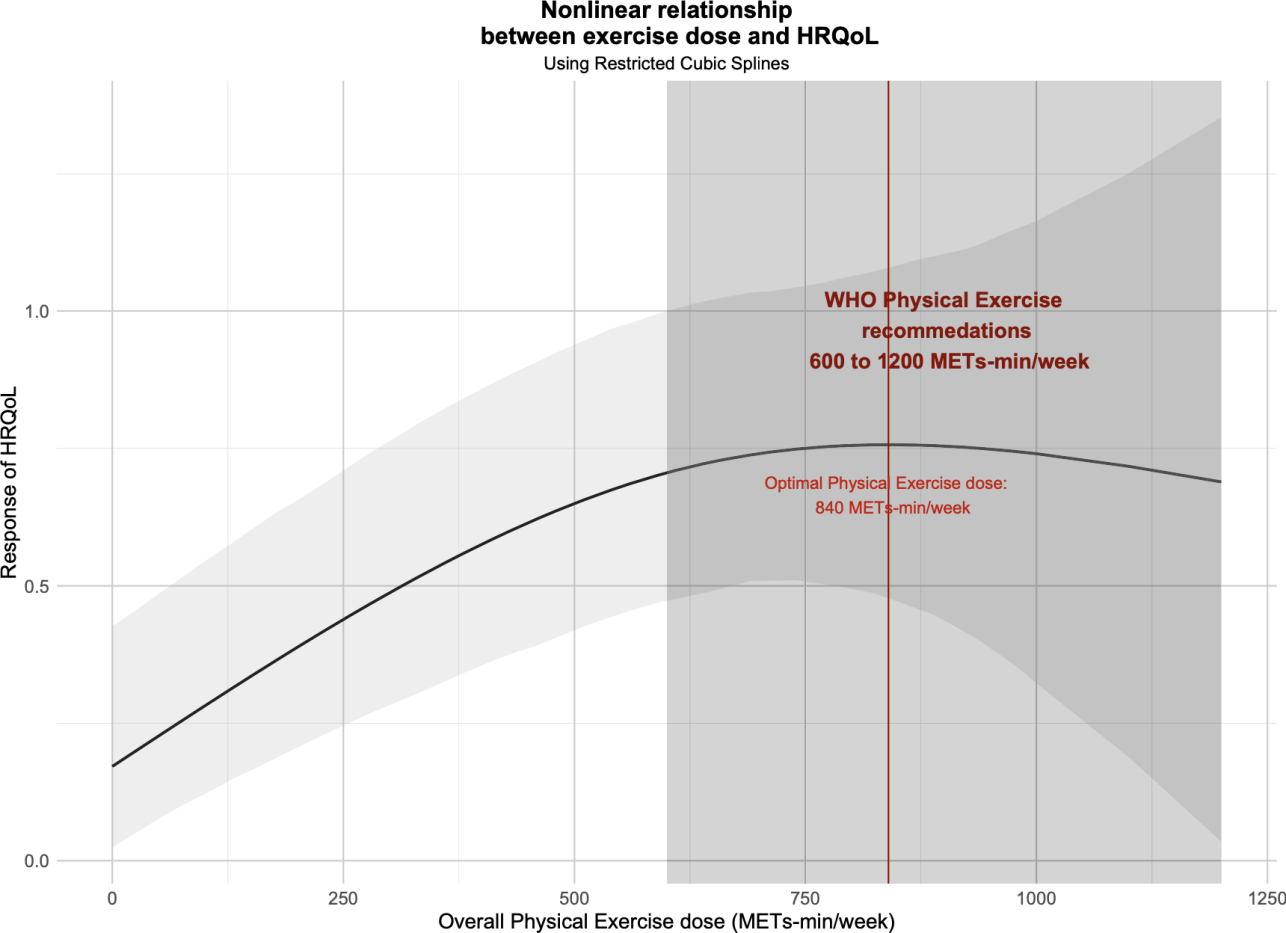
Dose-response relationship between overall exercise dose and change in the HRQoL of cancer survivors.

**Figure 3** shows the non-linear dose-response relationship between overall exercise dose and cancer survivors’ HRQoL. Notably, we found an inverted U-shape between the overall exercise dose and cancer survivors’ HRQoL, and an optimal peak dose for improving HRQoL existed. Overall exercise was found to be effective until 1100 METs-min/week (SMD: 0.716, 95% Crl: 0.169 to 1.280) but the optimal overall exercise dose was found to be 850 METs-min/week (SMD: 0.753, 95% Crl: 0.463 to 1.096).

**Figure 4:**
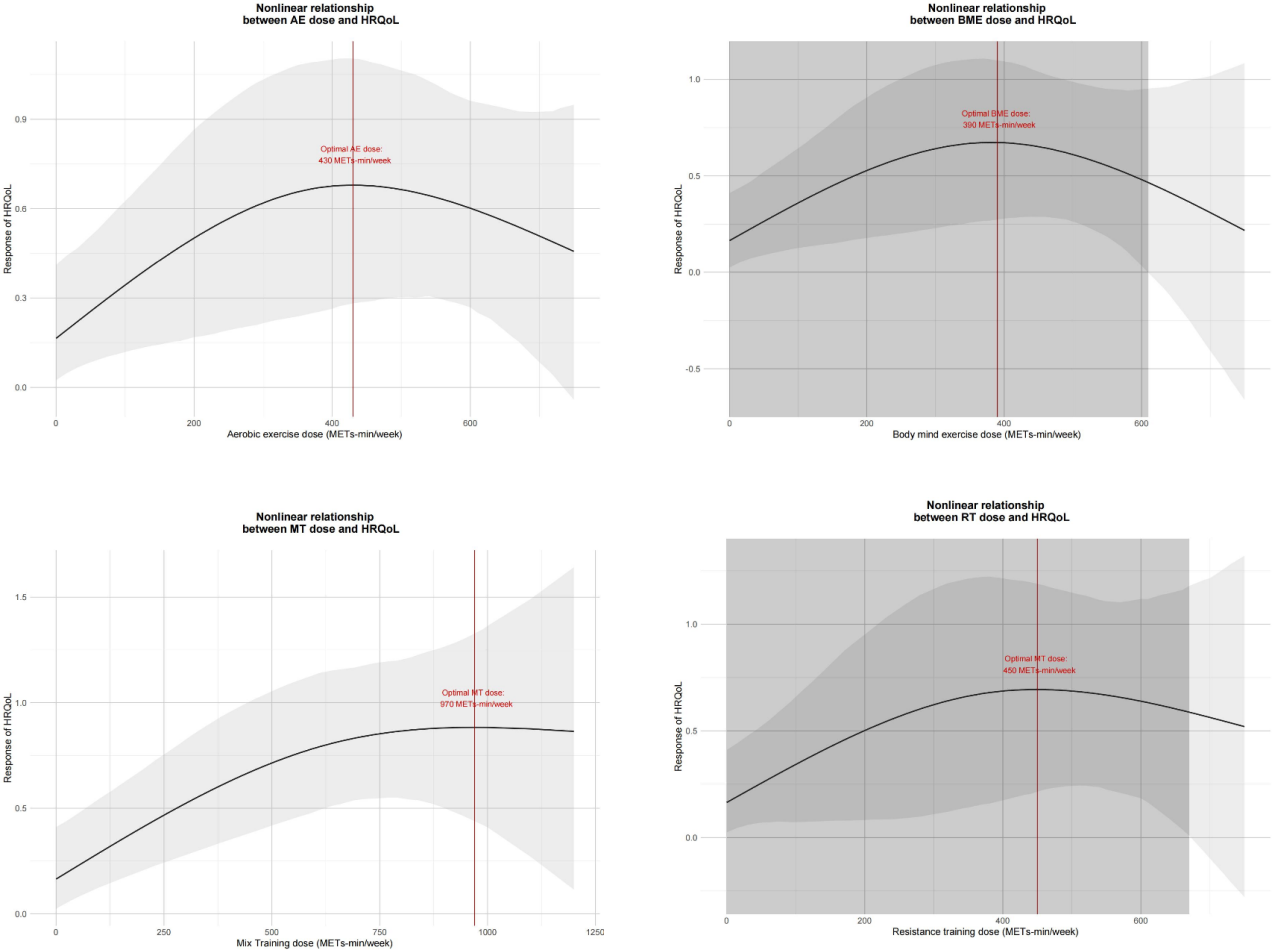
Dose-response relationship between different exercise doses and change in the HRQoL of cancer survivors. AE, aerobic exercise; RT, resistance training; MBE, mind-body exercise; MT, mixed training (aerobic + resistance Training).

**Figure 4** shows the non-linear dose-response relationship between different exercise doses and cancer survivors’ HRQoL in 18 studies involving 657 cancer survivors who participated in MT. The graphs show a trend of corresponding improvement in HRQoL with increasing MT dose until 1200 METs-min/week. The optimal MT dose was found to be 970 METs-min/week (SMD: 0.883, 95% Crl: 0.455 to 1.345).

Furthermore, in 16 studies involving 369 cancer survivors who participated in AE, in 7 studies involving 288 cancer survivors who participated in RT, and in 13 studies involving 311 cancer survivors who participated in MBE, we found the inverted U-shape for the AE, MBE, and RT intervention doses and cancer survivors’ HRQoL. None of these exercise doses exceeded 750 METs-min/week. AE was effective until 720 METs-min/week, and the optimal AE dose was found to be 430 METs-min/week (SMD: 0.681, 95% Crl: 0.206 to 1.099). Additionally, MBE was effective until 610 METs-min/week and the optimal MBE dose was found to be 390 METs-min/week (SMD: 0.672, 95% Crl: 0.259 to 1.087). The RT was effective until 660 METs-min/week, and the optimal RT dose was found to be 450 METs-min/week (SMD: 0.695, 95% Crl: 0.227 to 1.203).

### 3.4 Quality assessment of evidence and risk of bias

**Figure 5:**
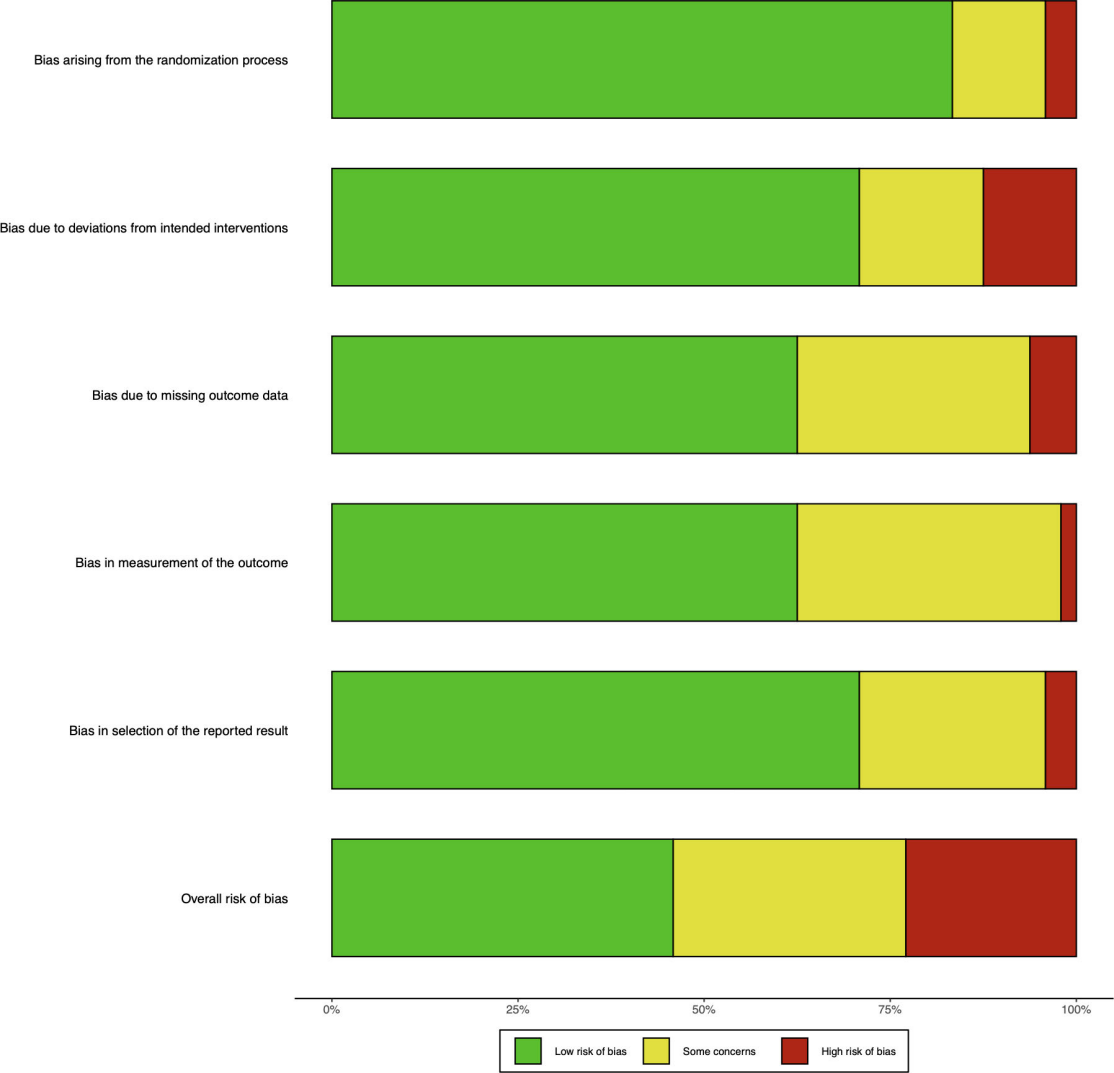
Cochrane risk of bias tool.

In total, 22 studies (46%) were found to have a low risk of bias, 15 studies (31%) were found to have an unclear risk of bias, and 11 studies (23%) were found to have a high risk of bias. **Figure 5** shows the result of the Cochrane Risk of Bias Tool and the study-level risk of bias assessments are presented in **Supplementary File 6**.

**Figure 6:**
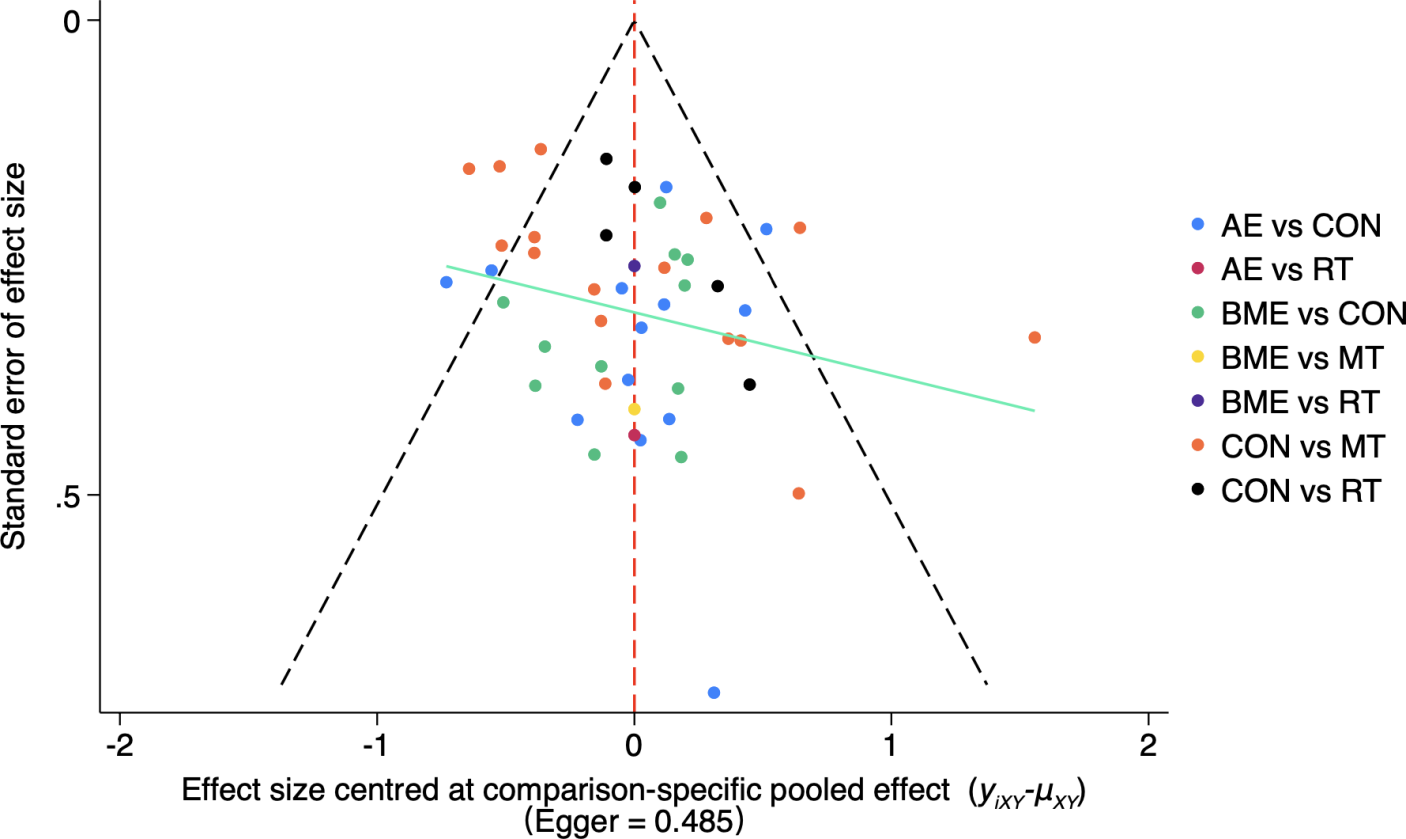
Funnel plot of the publication bias among the comparison groups. The funnel plot shows the relationship between the effect size and standard error of different exercise methods (AE, MBE, CON, RT, MT). The Egger’s test result was 0.485, indicating that the possibility of publication bias was small. AE, aerobic exercise; RT, resistance training; MBE, mind-body exercise; MT, mixed training (aerobic + resistance Training); CON, control.

The heterogeneity of HRQoL was moderate [I^2 = 58.9% (43.0%; 70.4%)] and the SIDE test of all outcomes showed that there was a percentage of comparisons with evidence of inconsistency (**Supplementary File 7**). At the same time, our funnel plot results showed that there was no publication bias and the funnel plot was symmetrical (**Figure 6**). In addition, we used Egger’s test to assess publication bias more formally. As shown in the figure, our included studies did not violate these two points.

### 3.5 Sensitivity analysis

We excluded 11 articles with a high risk of bias and reanalyzed the data. The results showed that the optimal dose of overall exercise was estimated to be 730 METs-min/week (SMD:0.727, 95%Crl: 0.461 to 1.038), 890 METs-min/week for MT (SMD:0.8418, 95%Crl: 0.226 to 1.306), 410 METs-min/week for AE (SMD:0.914, 95%Crl: 0.32 to 1.506), 410 METs-min/week for MBE (SMD:0.625, 95%Crl: 0.219 to 1.071), and 630 METs-min/week for RT (SMD:0.588, 95%Crl: 0.301 to 1.195). Additionally, these results of sensitive analysis confirmed the robustness of our study results. We provide visualization curve results in **Supplementary Figures 1**, **2** in **Supplementary File 9**.

## 4 Discussion

### 4.1 Main findings

Our systematic review and network meta-analysis included 48 randomized controlled trials involving 3050 cancer survivors. We determined the optimal exercise dose for overall exercise for improving cancer patients’ HRQoL, and as the dose increased, the effect took on an inverted U shape. We found that the optimal overall exercise dose to alter HRQoL in cancer survivors was 850 METs-min/week. Furthermore, in terms of different exercises, we found MT to be the best form of exercise to improve HRQoL, and we also found the optimal dose of MT (970 METs-min/week). Finally, we found that the optimal dose for improving HRQoL was 430 METs-min/week, 390 METs-min/week, and 450 METs-min/week for AE, MBE, and RT, respectively.

### 4.2 Strengths

First, we demonstrated that there is an optimal overall exercise dose. This is consistent with previous research that showed that exercise, no matter what it is, may not only be “healthy” but may actually have therapeutic effects (37). It was also well established in a cohort study that cancer survivors who engaged in 15 MET-hours of physical activity per week had a 27% lower risk of cancer-related mortality (38).

Notably, research suggests that exercise may exert its beneficial effects on cancer through various mechanisms, such as the alteration of circulating factors such as myokines and hormones released by skeletal muscle (39). One study in particular delved into these mechanisms, shedding light on how exercise can inhibit cancer cell proliferation, induce apoptosis, regulate metabolism, and enhance the immune environment (40). In our study, we determined the effective and safe value range of the overall exercise dose, which is undoubtedly a highlight for exercise and tumor research.

Furthermore, we found that the optimal form was MT (combined aerobic exercise and resistance training) for improving HRQoL in cancer survivors. The optimal MT dose was 970 METs-min/week. This finding not only aligns with previous analyses that unanimously recognize MT as the most effective form of exercise for enhancing HRQoL but also corresponds with international statements regarding exercise guidelines for cancer survivors (13, 41). In the included studies, MT consists of aerobic exercise and resistance training. In our study, we determined the optimal dose for resistance training to be 450 METs-min/week. Previous studies have indicated that resistance training correlates with a decreased risk of all-cause mortality, cardiovascular disease, and cancer-specific mortality (42). Furthermore, recent reviews of study outcomes suggest that non-traditional resistance training can yield equivalent or greater improvements in muscle strength, hypertrophy, body function, and composition compared to traditional methods. Studies on eccentric, cluster, and blood flow restriction training in older adults and other clinical populations provide robust evidence supporting its potential application in cancer research (43). This underscores the significance of integrating resistance exercise into the treatment regimen for cancer patients, as our study unequivocally confirms its efficacy in enhancing HRQoL within this demographic. Notably, our research identified the optimal intensity for resistance exercise, marking a substantial advancement over prior studies. Moreover, the optimal aerobic exercise dose to improve HRQoL was found to be 430 METs-min/week. We explored the main potential mechanism: an animal study has demonstrated that exercise is linked to the mobilization and redistribution of natural killer (NK) cells through adrenaline and interleukin-6 (IL-6), ultimately regulating tumorigenesis. This research result suggests that aerobic exercise can reduce the risk of cancer and disease recurrence by over 60% (44). Our research categorizes tai chi, qigong, and yoga as MBE (45). We found that the optimal dose of MBE was 390 METs-min/week. A previous study confirmed an improvement in the HRQoL of cancer patients through tai chi and qigong (46). On this basis, we determined the dose of MBE. However, the underlying mechanisms by which mind-body exercise improves symptoms in cancer patients are uncertain.

In addition, we conducted heterogeneity, inconsistency, and publication bias tests through network meta-analysis. Our results did not find any statistical differences in our data. We found that the heterogeneity was moderate and used a random effects model to merge the data. These key steps also indirectly boosted the credibility of our research from statistics to practice.

### 4.3 Limitations

While our study provides valuable insights, it is important to acknowledge its limitations. First, we did not conduct meticulous statistical heterogeneity analysis to explore possible heterogeneity among the studies.

Furthermore, in the preliminary analysis, we found that MT was the most effective in improving the HRQoL of cancer survivors. However, after sensitivity analysis, AE showed a more significant effect after excluding the studies with a high risk of bias. This change in results may reflect the impact of the risk of bias in different studies on the preliminary results. Specifically, some studies with high bias may have overestimated the effect of MT, causing the preliminary analysis results to favor MT.

This suggests that in high-quality, low-bias studies, AE may have an advantage in improving health-related quality of life. This finding reminds us that study quality and risk of bias should be considered when selecting interventions, and suggests that the relative effectiveness of different types of exercise may need to be re-evaluated in different research scenarios.

Nevertheless, this does not negate the potential value of MT, especially in specific patient groups. However, based on the results of the sensitivity analysis, AE may be a more robust and widely applicable option. Future studies should further explore the effects of these interventions in different cancer survivor groups to determine the optimal treatment plan.

Additionally, we did not report basic information regarding patient medication use, and it is plausible that patients received treatment not solely due to exercise-related improvements in outcomes. Moreover, our findings focused exclusively on the HRQoL of cancer patients and may not extend to all symptoms associated with cancer. We also note that the generalizability of our results may be limited as we observed an insufficient number of randomized controlled studies conducted in Asian populations. Although most exercise doses were reported following extensive deliberations between two reviewers, there remains the possibility of some misclassification at the dose level. Therefore, approximations were employed for dose characterization. Another potential limitation is the inclusion of cancer survivors with comorbidities (e.g., major depression or cognitive impairment) that may have underlying factors that may affect quality of life. These comorbidities may have had an impact on the intervention effect and may have confounded the relationship between exercise dose and HRQoL improvement to some extent. Although excluding these individuals would have resulted in a more homogeneous sample and a clearer understanding of the optimal exercise dose, we chose to include them in the study to increase the external validity of the study. Many cancer survivors face multiple health challenges simultaneously, and including these individuals made our findings more generalizable. Future studies may consider stratifying participants according to specific comorbidities or controlling for these variables in the study design to more precisely assess the effects of the exercise intervention. Finally, there may be possible differences in diagnosis stage and cancer type due to a higher proportion of female participants in the sample as certain cancer types (such as breast cancer) are more common in women, resulting in a relatively large number of female cancer survivors. This gender imbalance may have had an impact on the research results as a result of differences in physiology, psychology, and response to exercise intervention. Therefore, the inconsistent diagnosis of cancer patients in our study and the mixture of various cancer types may lead to doubts about the reliability of our results. In future RCT experiments, we should actively verify the true and effective value of our core research results, and provide recommended guidelines for exercise prescription for the next RCT experiment.

## 5 Conclusion

Our systematic review and dose-response network meta-analysis were conducted using the latest Bayesian modeling “MBNMA dose” and “ragjs” packages. We revealed a non-linear dose-response relationship between overall exercise dosage and health-related quality of life enhancement in cancer survivors. Notably, an inverted U-shaped pattern was observed, indicating an optimal peak dosage for HRQoL improvement. Effective exercise dosages ranged up to 1100 METs-min/week, with the optimal dosage identified as 850 METs-min/week. Additionally, MT was the optimal exercise form, and peak HRQoL improvement was observed at 970 METs-min/week. Across various exercise types, including AE, RT, and MBE, an inverted U-shaped relationship with HRQoL was evident, with optimal dosages identified for each modality. These findings provide essential insights for tailoring exercise prescriptions to optimize HRQoL outcomes among cancer survivors.

## Data Availability

The original contributions presented in the study are included in the article/**Supplementary Material**. Further inquiries can be directed to the corresponding author.
