# Supplementary material for: Optimal exercise dose-response improves health-related quality of life in cancer survivors: a systematic review and Bayesian network meta-analysis of RCTs
Source: Front Oncol. 2024 Dec 16;14:1510578. doi: 10.3389/fonc.2024.1510578 (PMC11682983; doi:10.3389/fonc.2024.1510578)
Supplement: Supplementary file 1 [file DataSheet1.docx]

Supplementary Material

[1 Supplementary File 1：Search strategy. 3](#_Toc179706631)

[2 Supplementary File 2：List of included studies. 12](#_Toc179706632)

[3 Supplementary File 3: Exercise Code. 17](#_Toc179706633)

[4 Supplementary File 4: Three key assumptions of network meta-analysis. 28](#_Toc179706634)

[4.1 Consistency 28](#_Toc179706635)

[Supplementary File 4 Table 1: Consistent and UME models fit comparison 28](#_Toc179706636)

[4.2 Transitivity 29](#_Toc179706637)

[Supplementary File 4 Figure 1: Validation Model Consistency Scatterplot 29](#_Toc179706638)

[4.2.1 Supplementary File 4 Table 2. Comparison of transitivity. 30](#_Toc179706639)

[5 Supplementary File 5：Nonlinear functions and models fit comparison. 34](#_Toc179706640)

[Supplementary File 5 Figure 1. “Split” NMA of overall exercise. 34](file:///C:\Users\Administrator\Desktop\Supplementary_Material.docx#_Toc179706641)

[Supplementary File 5 Figure 2. “Split” NMA of different exercises. 35](file:///C:\Users\Administrator\Desktop\Supplementary_Material.docx#_Toc179706642)

[Table 5.1: Models fit comparison. 36](#_Toc179706643)

[Supplementary File 5 Figure 3. Deviance box plot at overall exercise level. 38](file:///C:\Users\Administrator\Desktop\Supplementary_Material.docx#_Toc179706644)

[Supplementary File 5 Figure 4. Deviance box plot at different exercise levels. 39](file:///C:\Users\Administrator\Desktop\Supplementary_Material.docx#_Toc179706645)

[6 Supplementary File 6: Study level Risk of Bias analysis. 40](#_Toc179706646)

[Supplementary File 6 Figure 9: Study level Risk of Bias analysis 40](file:///C:\Users\Administrator\Desktop\Supplementary_Material.docx#_Toc179706647)

[7 Supplementary File 7: Inconsistency and heterogeneity. 41](#_Toc179706648)

[8 Supplementary File 8: Exercise programs Definitions. 46](#_Toc179706649)

[47](#_Toc179706650)

[Supplementary File 9 Figure 1: Sensitivity analysis of overall exercise dose-response relationship curve. 47](#_Toc179706651)

[Supplementary File 9 Figure 2: Sensitivity analysis of different exercise dose-response relationship curves. 48](#_Toc179706652)

[Reference 48](#_Toc179706653)

# Supplementary File 1：Search strategy.

| 1.PubMed  (2023-12-1) | 1. neoplasms [MEsH Terms]  2.Cancer[Title/Abstract]  3.Tumour[Title/Abstract]  4.Neoplasm[Title/Abstract]  5.Oncology[Title/Abstract]  6.Carcinomatosis[Title/Abstract]  7.Radiotherapy[Title/Abstract]  8.Chemotherapy[Title/Abstract]  9.Targeted therapy[Title/Abstract]  10.Immunotherapy[Title/Abstract]  11.#1 or #2 or #3 or #4 or #5 or #6 or #7 or #8 or #9 or #10(4747363)  12.exercise[MEsH Terms]  13.physical activity[Title/Abstract]  14.physical[Title/Abstract]  15.aerobic exercise[Title/Abstract]  16.resistance exercise[Title/Abstract]  17.strength exercise[Title/Abstract]  18.flexibility exercise[Title/Abstract]  19.balance exercise[Title/Abstract]  20.walking[Title/Abstract]  21.isometric training[Title/Abstract]  22.plyometric exercise[Title/Abstract]  23.isotonic training[Title/Abstract]  24.isodynamic training[Title/Abstract]  25.isokinetic training[Title/Abstract]  26.Taichi[Title/Abstract]  27.Taiji[Title/Abstract]  28.Baduanjin[Title/Abstract]  29.Yoga[Title/Abstract]  30.Dance[Title/Abstract]  31.Running[Title/Abstract]  32.Qigong[Title/Abstract]  33.#12 or #13 or #14 or #15 or #16 or #17 or #18 or #19 or #20 or #21or #22 or #23 or #24 or #25 or #26or #26 or #27 or #28 or #29 or #30or #31(1172025)  34. quality of life [Title/Abstract] or QoL [Title/Abstract] or life quality [Title/Abstract] or living quality [Title/Abstract] or health-related quality of life [Title/Abstract] or quantity life [Title/Abstract] (393348)  35. control trial or RCT or RCTs or Randomized controlled trial or randomized controlled trial(190249)  #11 and #33 and #34 and #35 1170 |
| --- | --- |
| 2. Web of Science  (2023-12-1) | 1.neoplasms.tw  2.Cancer.tw  3.Tumour.tw  4.Neoplasm.tw  5.Oncology.tw  6.Carcinomatosis.tw  7.Radiotherapy.tw  8.Chemotherapy.tw  9.Targeted therapy.tw  10.Immunotherapy.tw  11.#1 or #2 or #3 or #4 or #5 or #6or #7or #8 or #9 or #10(4739652)  12.exercise.tw  13.physical activity.tw  14.physical.tw  15.aerobic exercise.tw  16.resistance exercise.tw  17.strength exercise.tw  18.flexibility exercise.tw  19.balance exercise.tw  20.walking.tw  21.isometric training.tw  22.plyometric exercise.tw  23.isotonic training.tw  24.isodynamic training.tw  25.isokinetic training.tw  26.Taichi.tw  27.Taiji.tw  28.Baduanjin.tw  29.Yoga.tw  30.Dance.tw  31.Running.tw  32.Qigong.tw  33.#12 or #13 or #14 or #15 or #16 or #17 or #18 or #19 or #20 or #21or #22 or #23 or #24 or #25 or #26or #26 or #27 or #28 or #29 or #30or #31(3287004)  34.quality of life.tw or QoL.tw or life quality.tw or living quality.tw or health-related quality of life.tw or quantity life.tw (794008)  35.control trial.tw or RCT.tw or RCTs.tw or Randomized controlled trial.tw or randomized controlled trial.tw (825862)  #11 and #33 and #34 and #35 6113 |
| 3. The Cochrane Library  (2023-12-1) | Neoplasms [MeSH tree]  1.neoplasms  2.Cancer  3.Tumour  4.Neoplasm  5.Oncology  6.Carcinomatosis  7.Radiotherapy  8.Chemotherapy  9.Targeted therapy  10.Immunotherapy  11.#1 or #2 or #3 or #4 or #5 or #6or #7 or #8 or #9 or #10(332331)  12.exercise [MeSH tree]  13.physical activity  14.physical  15.aerobic exercise  16.resistance exercise  17.strength exercise  18.flexibility exercise  19.balance exercise  20.walking  21.isometric training  22.plyometric exercise  23.isotonic training  24.isodynamic training  25.isokinetic training  26.Taichi  27.Taiji  28.Baduanjin  29.Yoga  30.Dance  31.Running  32.Qigong  33.#12 or #13 or #14 or #15 or #16 or #17 or #18 or #19 or #20 or #21or #22 or #23 or #24 or #25 or #26or #26 or #27 or #28 or #29 or #30or #31 (238931)  34.quality of life or QoL or life quality or living quality or health-related quality of life or quantity life(167707)  35.control trial or RCT or RCTs or Randomized controlled trial or randomized controlled trial (902962)  #11 and #33 and #34 and #35(7108) |
| 4. Embase  (2023-12-1) | ‘Neoplasm’/exp  1. neoplasms:ab.ti.kw  2. Cancer:ab.ti.kw  3. Tumour:ab.ti.kw  4. Neoplasm:ab.ti.kw  5. Oncology:ab.ti.kw  6.Carcinomatosis:ab.ti.kw  7.Radiotherapy:ab.ti.kw  8.Chemotherapy:ab.ti.kw  9.Targeted therapy:ab.ti.kw  10.Immunotherapy:ab.ti.kw  11.#1 or #2 or #3 or #4 or #5 or #6or #7 or #8 or #9 or #10(6892610)  12.‘exercise’/exp  13.physical activity:ab.ti.kw  14.physical:ab.ti.kw  15.aerobic exercise:ab.ti.kw  16.resistance exercise:ab.ti.kw  17.strength exercise:ab.ti.kw  18.flexibility exercise:ab.ti.kw  19.balance exercise:ab.ti.kw  20.walking:ab.ti.kw  21.isometric training:ab.ti.kw  22.plyometric exercise:ab.ti.kw  23.isotonic training:ab.ti.kw  24.isodynamic training:ab.ti.kw  25.isokinetic training:ab.ti.kw  26.Taichi:ab.ti.kw  27.Taiji:ab.ti.kw  28.Baduanjin:ab.ti.kw  29.Yoga:ab.ti.kw  30.Dance:ab.ti.kw  31.Running:ab.ti.kw  32.Qigong:ab.ti.kw  33.#12 or #13 or #14 or #15 or #16 or #17 or #18 or #19 or #20 or #21or #22 or #23 or #24 or #25 or #26or #26 or #27 or #28 or #29 or #30or #31(170730)  34.quality of life:ab.ti.kw or QoL:ab.ti.kw or life quality or living quality:ab.ti.kw or health-related quality of life:ab.ti.kw or quantity life:ab.ti.kw (614567)  35.control trial:ab.ti.kw or RCT:ab.ti.kw or RCTs:ab.ti.kw or Randomized controlled trial:ab.ti.kw or randomized controlled trial:ab.ti.kw (263136)  #11 and #33 and #34 and #35 (1961) |
| 5. SCOPUS  (2023-12-1) | 1.neoplasms[‘TITLE-ABS-KEY’]  2.Cancer[‘TITLE-ABS-KEY’]  3.Tumour[‘TITLE-ABS-KEY’]  4.Neoplasm[‘TITLE-ABS-KEY’]  5.Oncology[‘TITLE-ABS-KEY’]  6.Carcinomatosis[‘TITLE-ABS-KEY’]  7.Radiotherapy[‘TITLE-ABS-KEY’]  8.Chemotherapy[‘TITLE-ABS-KEY’]  9.Targeted therapy[‘TITLE-ABS-KEY’]  10.Immunotherapy[‘TITLE-ABS-KEY’]  11.#1 or #2 or #3 or #4 or #5 or #6or #7 or #8 or #9 or #10(6222702)  12.exercise[‘TITLE-ABS-KEY’]  13.physical activity[‘TITLE-ABS-KEY’]  14.physical[‘TITLE-ABS-KEY’]  15.aerobic exercise[‘TITLE-ABS-KEY’]  16.resistance exercise[‘TITLE-ABS-KEY’]  17.strength exercise[‘TITLE-ABS-KEY’]  18.flexibility exercise[‘TITLE-ABS-KEY’]  19.balance exercise[‘TITLE-ABS-KEY’]  20.walking[‘TITLE-ABS-KEY’]  21.isometric training[‘TITLE-ABS-KEY’]  22.plyometric exercise[‘TITLE-ABS-KEY’]  23.isotonic training[‘TITLE-ABS-KEY’]  24.isodynamic [‘TITLE-ABS-KEY’]  25.isokinetic training[‘TITLE-ABS-KEY’]  26.Taichi[‘TITLE-ABS-KEY’]  27.Taiji[‘TITLE-ABS-KEY’]  28.Baduanjin[‘TITLE-ABS-KEY’]  29.Yoga[‘TITLE-ABS-KEY’]  30.Dance[‘TITLE-ABS-KEY’]  31.Running[‘TITLE-ABS-KEY’]  32.Qigong[‘TITLE-ABS-KEY’]  33.#12 or #13 or #14 or #15 or #16 or #17 or #18 or #19 or #20 or #21or #22 or #23 or #24 or #25 or #26or #26 or #27 or #28 or #29 or #30or #31(10447351)  34. quality of life or QoL or life quality or living quality or health-related quality of life or quantity life (3747383)  35. control trial or RCT or RCTs or Randomized controlled trial or randomized controlled trial (17736953)  #11 and #33 and #34 and #35 (2219) |

# Supplementary File 2：List of included studies.

1. Courneya, K. S., Mackey, J. R., Bell, G. J., Jones, L. W., Field, C. J., & Fairey, A. S. (2003). Randomized controlled trial of exercise training in postmenopausal breast cancer survivors: cardiopulmonary and quality of life outcomes. *Journal of clinical oncology*, *21*(9), 1660-1668.
2. Courneya, K. S., Friedenreich, C. M., Quinney, H. A., Fields, A. L. A., Jones, L. W., & Fairey, A. S. (2003). A randomized trial of exercise and quality of life in colorectal cancer survivors. *European journal of cancer care*, *12*(4), 347-357.
3. Ohira, T., Schmitz, K. H., Ahmed, R. L., & Yee, D. (2006). Effects of weight training on quality of life in recent breast cancer survivors: the Weight Training for Breast Cancer Survivors (WTBS) study. *Cancer: Interdisciplinary International Journal of the American Cancer Society*, *106*(9), 2076-2083.
4. Nicole Culos‐Reed, S., Carlson, L. E., Daroux, L. M., & Hately‐Aldous, S. (2006). A pilot study of yoga for breast cancer survivors: physical and psychological benefits. *Psycho‐Oncology: Journal of the Psychological, Social and Behavioral Dimensions of Cancer*, *15*(10), 891-897.
5. Chandwani, K. D., Perkins, G., Nagendra, H. R., Raghuram, N. V., Spelman, A., Nagarathna, R., ... & Cohen, L. (2014). Randomized, controlled trial of yoga in women with breast cancer. *Journal of Clinical Oncology*, *32*(10), 1058.
6. Milne, H. M., Wallman, K. E., Gordon, S., & Courneya, K. S. (2008). Effects of a combined aerobic and resistance exercise program in breast cancer survivors: a randomized controlled trial. *Breast cancer research and treatment*, *108*, 279-288.
7. Lee, S. A., Kang, J. Y., Kim, Y. D., An, A. R., Kim, S. W., Kim, Y. S., & Lim, J. Y. (2010). Effects of a scapula-oriented shoulder exercise programme on upper limb dysfunction in breast cancer survivors: a randomized controlled pilot trial. *Clinical rehabilitation*, *24*(7), 600-613.
8. Sprod, L. K., Janelsins, M. C., Palesh, O. G., Carroll, J. K., Heckler, C. E., Peppone, L. J., ... & Mustian, K. M. (2012). Health-related quality of life and biomarkers in breast cancer survivors participating in tai chi chuan. *Journal of Cancer Survivorship*, *6*, 146-154.
9. Littman, A. J., Bertram, L. C., Ceballos, R., Ulrich, C. M., Ramaprasad, J., McGregor, B., & McTiernan, A. (2012). Randomized controlled pilot trial of yoga in overweight and obese breast cancer survivors: effects on quality of life and anthropometric measures. *Supportive Care in Cancer*, *20*, 267-277.
10. Siedentopf, F., Utz-Billing, I., Gairing, S., Schoenegg, W., Kentenich, H., & Kollak, I. (2013). Yoga for patients with breast cancer and its impact on quality of life–a randomized controlled trial. *Geburtshilfe und Frauenheilkunde*, *73*(04), 311-317.
11. Pinto, B. M., Papandonatos, G. D., Goldstein, M. G., Marcus, B. H., & Farrell, N. (2013). Home‐based physical activity intervention for colorectal cancer survivors. *Psycho‐oncology*, *22*(1), 54-64.
12. Broderick, J. M., Guinan, E., Kennedy, M. J., Hollywood, D., Courneya, K. S., Culos-Reed, S. N., ... & Hussey, J. (2013). Feasibility and efficacy of a supervised exercise intervention in de-conditioned cancer survivors during the early survivorship phase: the PEACH trial. *Journal of Cancer Survivorship*, *7*, 551-562.
13. Streckmann, F., Kneis, S., Leifert, J. A., Baumann, F. T., Kleber, M., Ihorst, G., ... & Bertz, H. (2014). Exercise program improves therapy-related side-effects and quality of life in lymphoma patients undergoing therapy. *Annals of oncology*, *25*(2), 493-499.
14. Murtezani, A., Ibraimi, Z., Bakalli, A., Krasniqi, S., Disha, E. D., & Kurtishi, I. (2014). The effect of aerobic exercise on quality of life among breast cancer survivors: a randomized controlled trial. *Journal of cancer research and therapeutics*, *10*(3), 658-664.
15. Jensen, W., Baumann, F. T., Stein, A., Bloch, W., Bokemeyer, C., De Wit, M., & Oechsle, K. (2014). Exercise training in patients with advanced gastrointestinal cancer undergoing palliative chemotherapy: a pilot study. *Supportive Care in Cancer*, *22*, 1797-1806.
16. Chang, N. W., Lin, K. C., Lee, S. C., Chan, J. Y. H., Lee, Y. H., & Wang, K. Y. (2014). Effects of an early postoperative walking exercise programme on health status in lung cancer patients recovering from lung lobectomy. *Journal of Clinical Nursing*, *23*(23-24), 3391-3402.
17. Brocki, B. C., Andreasen, J., Nielsen, L. R., Nekrasas, V., Gorst-Rasmussen, A., & Westerdahl, E. (2014). Short and long-term effects of supervised versus unsupervised exercise training on health-related quality of life and functional outcomes following lung cancer surgery–a randomized controlled trial. *Lung Cancer*, *83*(1), 102-108.
18. Travier, N., Velthuis, M. J., Steins Bisschop, C. N., van den Buijs, B., Monninkhof, E. M., Backx, F., ... & May, A. M. (2015). Effects of an 18-week exercise programme started early during breast cancer treatment: a randomised controlled trial. *BMC medicine*, *13*(1), 1-11.
19. Van Vulpen, J. K., Velthuis, M. J., Steins Bisschop, C. N., Travier, N., Van Den Buijs, B. J., Backx, F. J., ... & May, A. M. (2016). Effects of an exercise program in colon cancer patients undergoing chemotherapy. *Med Sci Sports Exerc*, *48*(5), 767-775.
20. Rossi, A., Garber, C. E., Ortiz, M., Shankar, V., Goldberg, G. L., & Nevadunsky, N. S. (2016). Feasibility of a physical activity intervention for obese, socioculturally diverse endometrial cancer survivors. *Gynecologic Oncology*, *142*(2), 304-310.
21. De Luca, V., Minganti, C., Borrione, P., Grazioli, E., Cerulli, C., Guerra, E., ... & Parisi, A. (2016). Effects of concurrent aerobic and strength training on breast cancer survivors: a pilot study. *Public health*, *136*, 126-132.
22. Vanderbyl, B. L., Mayer, M. J., Nash, C., Tran, A. T., Windholz, T., Swanson, T., ... & Jagoe, R. T. (2017). A comparison of the effects of medical Qigong and standard exercise therapy on symptoms and quality of life in patients with advanced cancer. *Supportive Care in Cancer*, *25*, 1749-1758.
23. Pisu, M., Demark-Wahnefried, W., Kenzik, K. M., Oster, R. A., Lin, C. P., Manne, S., ... & Martin, M. Y. (2017). A dance intervention for cancer survivors and their partners (RHYTHM). *Journal of Cancer Survivorship*, *11*, 350-359.
24. Strunk, M. A., Zopf, E. M., Steck, J., Hamacher, S., Hallek, M., & Baumann, F. T. (2018). Effects of kyusho jitsu on physical activity-levels and quality of life in breast cancer patients. *in vivo*, *32*(4), 819-824.
25. Leclerc, A. F., Slomian, J., Jerusalem, G., Coucke, P., Bury, T., Deflandre, D., ... & Maquet, D. (2018). Exercise and education program after breast cancer: benefits on quality of life and symptoms at 3-, 6-, 12-, and 24-months’ follow-up. *Clinical Breast Cancer*, *18*(5), e1189-e1204.
26. Dieli-Conwright, C. M., Courneya, K. S., Demark-Wahnefried, W., Sami, N., Lee, K., Sweeney, F. C., ... & Mortimer, J. E. (2018). Aerobic and resistance exercise improves physical fitness, bone health, and quality of life in overweight and obese breast cancer survivors: a randomized controlled trial. *Breast Cancer Research*, *20*(1), 1-10.
27. Cuthbert, C. A., King-Shier, K. M., Ruether, J. D., Tapp, D. M., Wytsma-Fisher, K., Fung, T. S., & Culos-Reed, S. N. (2018). The effects of exercise on physical and psychological outcomes in cancer caregivers: Results from the RECHARGE randomized controlled trial. *Annals of Behavioral Medicine*, *52*(8), 645-661.
28. Villumsen, B. R., Jorgensen, M. G., Frystyk, J., Hørdam, B., & Borre, M. (2019). Home‐based ‘exergaming’was safe and significantly improved 6‐min walking distance in patients with prostate cancer: a single‐blinded randomised controlled trial. *BJU international*, *124*(4), 600-608.
29. Pasyar, N., Tashnizi, N. B., Mansouri, P., & Tahmasebi, S. (2019). Effect of yoga exercise on the quality of life and upper extremity volume among women with breast cancer related lymphedema: A pilot study. *European Journal of Oncology Nursing*, *42*, 103-109.
30. Mijwel, S., Jervaeus, A., Bolam, K. A., Norrbom, J., Bergh, J., Rundqvist, H., & Wengström, Y. (2019). High-intensity exercise during chemotherapy induces beneficial effects 12 months into breast cancer survivorship. *Journal of Cancer Survivorship*, *13*, 244-256.
31. Christensen, J. F., Sundberg, A., Osterkamp, J., Thorsen-Streit, S., Nielsen, A. B., Olsen, C. K., ... & Hojman, P. (2019). Interval walking improves glycemic control and body composition after cancer treatment: a randomized controlled trial. *The Journal of Clinical Endocrinology & Metabolism*, *104*(9), 3701-3712.
32. Cešeiko, R., Eglītis, J., Srebnijs, A., Timofejevs, M., Purmalis, E., Erts, R., ... & Tomsone, S. (2019). The impact of maximal strength training on quality of life among women with breast cancer undergoing treatment. *Exp Oncol*, *41*(2), 166-72.
33. Quist, M., Langer, S. W., Lillelund, C., Winther, L., Laursen, J. H., Christensen, K. B., ... & Adamsen, L. (2020). Effects of an exercise intervention for patients with advanced inoperable lung cancer undergoing chemotherapy: a randomized clinical trial. *Lung Cancer*, *145*, 76-82.
34. Hong, Y., Wu, C., & Wu, B. (2020). Effects of resistance exercise on symptoms, physical function, and quality of life in gastrointestinal cancer patients undergoing chemotherapy. *Integrative Cancer Therapies*, *19*, 1534735420954912.
35. Cunningham, E., Weaver, R. R., Lemonde, M., Dogra, S., & Nonoyama, M. L. (2020). Nordic pole walking for individuals with cancer: a feasibility randomized controlled trial assessing physical function and health-related quality of life. *Rehabilitation Oncology*, *38*(2), 81-91.
36. Mardani, A., Pedram Razi, S., Mazaheri, R., Haghani, S., & Vaismoradi, M. (2021). Effect of the exercise programme on the quality of life of prostate cancer survivors: A randomized controlled trial. *International Journal of Nursing Practice*, *27*(2), e12883.
37. Loughney, L., West, M. A., Kemp, G. J., Rossiter, H. B., Burke, S. M., Cox, T., ... & Jack, S. (2016). The effects of neoadjuvant chemoradiotherapy and an in-hospital exercise training programme on physical fitness and quality of life in locally advanced rectal cancer patients (The EMPOWER Trial): study protocol for a randomised controlled trial. *Trials*, *17*(1), 1-13.
38. Lin, K. Y., Cheng, H. C., Yen, C. J., Hung, C. H., Huang, Y. T., Yang, H. L., ... & Tsai, K. L. (2021). Effects of exercise in patients undergoing chemotherapy for head and neck cancer: a pilot randomized controlled trial. *International journal of environmental research and public health*, *18*(3), 1291.
39. Gal, R., Monninkhof, E. M., van Gils, C. H., Groenwold, R. H., Elias, S. G., van den Bongard, D. H., ... & May, A. M. (2021). Effects of exercise in breast cancer patients: implications of the trials within cohorts (TwiCs) design in the UMBRELLA Fit trial. *Breast Cancer Research and Treatment*, *190*(1), 89-101.
40. Eyigör, S., Apaydin, S., Yesil, H., Tanıgor, G., & Hopanci Bicakli, D. (2021). Effects of Yoga on Phase Angle and Quality of Life in Patients with Breast Cancer: A Randomized, Single-Blind, Controlled Trial. *Complementary Medicine Research*, *28*(6), 523-532.
41. Cheung, D. S. T., Takemura, N., Lam, T. C., Ho, J. C. M., Deng, W., Smith, R., ... & Lin, C. C. (2021). Feasibility of aerobic exercise and Tai-Chi interventions in advanced lung cancer patients: a randomized controlled trial. *Integrative Cancer Therapies*, *20*, 15347354211033352.
42. 59. Mikkelsen, M. K., Lund, C. M., Vinther, A., Tolver, A., Johansen, J. S., Chen, I., ... & Jarden, M. (2022). Effects of a 12-week multimodal exercise intervention among older patients with advanced cancer: results from a randomized controlled trial. *The oncologist*, *27*(1), 67-78.
43. Reljic, D., Herrmann, H. J., Jakobs, B., Dieterich, W., Mougiakakos, D., Neurath, M. F., & Zopf, Y. (2022). Feasibility, safety, and preliminary efficacy of very low-volume interval training in advanced cancer patients. *Medicine and Science in Sports and Exercise*, *54*(11), 1817.
44. Kaushik, D., Shah, P. K., Mukherjee, N., Ji, N., Dursun, F., Kumar, A. P., ... & Liss, M. A. (2022). Effects of yoga in men with prostate cancer on quality of life and immune response: a pilot randomized controlled trial. *Prostate cancer and prostatic diseases*, *25*(3), 531-538.
45. Machado, P., Pimenta, S., Garcia, A. L., Nogueira, T., Silva, S., Dos Santos, C. L., ... & Cruz, J. (2023). Effect of home-based exercise training on quality of life after lung cancer surgery: a multicenter randomized controlled trial. *Annals of Surgical Oncology*, 1-13.
46. Schmitt, J., Lindner, N., Reuss‐Borst, M., Holmberg, H. C., & Sperlich, B. (2016). A 3‐week multimodal intervention involving high‐intensity interval training in female cancer survivors: a randomized controlled trial. *Physiological reports*, *4*(3), e12693.
47. Park, S. H., Tish Knobf, M., & Jeon, S. (2019). Endocrine therapy–related symptoms and quality of life in female cancer survivors in the Yale Fitness Intervention Trial. *Journal of Nursing Scholarship*, *51*(3), 317-325.
48. Moadel, A. B., Shah, C., Wylie-Rosett, J., Harris, M. S., Patel, S. R., Hall, C. B., & Sparano, J. A. (2007). Randomized controlled trial of yoga among a multiethnic sample of breast cancer patients: effects on quality of life. *Journal of Clinical Oncology*, *25*(28), 4387-4395.

# Supplementary File 3: Exercise Code.

| **Study** | **Invention** | **Mean^change^** | **SE（standard error）** | **Mets-min** | **duration /week** | **MET-min/week** | **Exact dose** | **Residual dose** |
| --- | --- | --- | --- | --- | --- | --- | --- | --- |
| Kerry et.al 2003 | AE | 5.8 | 2.401041489 | 6.8 | 15 | 612 | 500 | 112 |
|  | CON | 0.5 | 1.905911935 | 0 | 0 | 0 | 0 | 0 |
| Courneya et.al 2004 | AE | 0.9 | 1.569672294 | 4 | 16 | 300 | 250 | 50 |
|  | CON | 2 | 2.617003891 | 0 | 0 | 0 | 0 | 0 |
| Tetsuya et.al 2006 | RT | 2.2 | 1.377381386 | 3.5 | 13 | 210 | 250 | -40 |
|  | CON | -0.7 | 1.434137365 | 0 | 0 | 0 | 0 | 0 |
| Kavita et.al 2014 | Yoga | 2.3 | 1.452583898 | 2.3 | 6 | 414 | 500 | -86 |
|  | CON | 0.2 | 1.15316257 | 2.3 | 6 | 414 | 500 | -86 |
| Helen et.al 2008 | MT | 15.8 | 1.895821087 | 3.5 | 12 | 525 | 500 | 25 |
|  | CON | -5.5 | 1.759016175 | 0 | 0 | 0 | 0 | 0 |
| Seung et.al 2010 | RT | 15.9 | 6.599999884 | 5 | 8 | 150 | 250 | -100 |
|  | CON | -3.3 | 4.5020982 | 0 | 0 | 0 | 0 | 0 |
| Lisa et.al 2012 | Taichi | 5.31 | 6.334263967 | 3 | 12 | 540 | 500 | 40 |
|  | CON | 2.76 | 5.435645395 | 0 | 0 | 0 | 0 | 0 |
| Alyson et.al 2012 | Yoga | 1.3 | 1.81968397 | 2.3 | 24 | 862.5 | 750 | 112.5 |
|  | CON | -0.1 | 2.625188145 | 0 | 0 | 0 | 0 | 0 |
| Siedentopf et.al 2013 | Yoga | 7.78 | 4.140161308 | 4 | 5 | 480 | 500 | -20 |
|  | CON | 4.94 | 4.817270127 | 0 | 0 | 0 | 0 | 0 |
| Bernardine et.al 2013 | AE | 1.6 | 2.854420154 | 4.8 | 12 | 288 | 250 | 38 |
|  | CON | -2.5 | 2.514013717 | 0 | 0 | 0 | 0 | 0 |
| Broderick et.al 2013 | AE | 3.8 | 2.87636543 | 3.5 | 8 | 315 | 250 | 65 |
|  | CON | 3.8 | 2.226993499 | 0 | 0 | 0 | 0 | 0 |
| Streckmann et.al 2014 | MT | 12 | 6.569890527 | 3.9 | 36 | 468 | 500 | -32 |
|  | CON | -1 | 5.4 | 0 | 0 | 0 | 0 | 0 |
| Ardiana et.al 2014 | AE | 9.1 | 1.51206261 | 4.8 | 10 | 504 | 500 | 4 |
|  | CON | -0.5 | 1.42247584 | 0 | 0 | 0 | 0 | 0 |
| Wiebke et.al 2014 | RT | 14.7 | 12.08729511 | 3.5 | 12 | 315 | 250 | 65 |
|  | AE | 13.3 | 5.005896604 | 4.3 | 12 | 387 | 250 | 137 |
| Chang et.al 2014 | Walking | 3 | 0.47470714 | 4.3 | 12 | 180.6 | 250 | -69.4 |
|  | CON | 0.37 | 0.450356756 | 0 | 0 | 0 | 0 | 0 |
| Barbara et.al 2014 | MT | 5.5 | 1.639904178 | 4.15 | 10 | 145.25 | 250 | -104.75 |
|  | CON | 3.75 | 1.564562924 | 0 | 0 | 0 | 0 | 0 |
| Noémie et.al 2015 | MT | 4.2 | 1.959821714 | 4 | 36 | 240 | 250 | -10 |
|  | CON | 5.5 | 2.325935366 | 0 | 0 | 0 | 0 | 0 |
| Jonna et.al 2016 | MT | 3.1 | 4.450066204 | 4 | 18 | 400 | 500 | -100 |
|  | CON | 1.1 | 5.086809 | 0 | 0 | 0 | 0 | 0 |
| Amerigo et.al 2016 | MT | 4 | 2.623423939 | 4 | 12 | 360 | 500 | -140 |
|  | CON | -1 | 2.886751346 | 0 | 0 | 0 | 0 | 0 |
| Luca et.al 2016 | MT | 13.2 | 4.056969178 | 4 | 24 | 560 | 500 | 60 |
|  | CON | -4.8 | 4.849433114 | 0 | 0 | 0 | 0 | 0 |
| Vanderbyl et.al 2017 | Qigong | 3.6 | 1.989974874 | 3 | 6 | 270 | 250 | 20 |
|  | MT | 3.5 | 3.910636383 | 4 | 6 | 360 | 500 | -140 |
| Maria et.al 2017 | Dance | 6.1 | 4.64567627 | 3 | 12 | 675 | 750 | -75 |
|  | CON | -2.2 | 4.624189 | 0 | 0 | 0 | 0 | 0 |
| Maximilian et.al 2018 | Taichi | 11.5 | 2.753412434 | 3 | 24 | 540 | 500 | 40 |
|  | CON | -2.75 | 4.234182323 | 0 | 0 | 0 | 0 | 0 |
| Anne et.al 2018 | MT | 16 | 2.557342325 | 4 | 12 | 1080 | 1000 | 80 |
|  | CON | 1 | 2.338928853 | 0 | 0 | 0 | 0 | 0 |
| Christina et.al 2018 | MT | 11.1 | 1.340969801 | 4 | 16 | 600 | 500 | 100 |
|  | CON | -0.3 | 1.279921879 | 0 | 0 | 0 | 0 | 0 |
| Colleen et.al 2018 | MT | 0.3 | 1.323352 | 4 | 12 | 720 | 750 | -30 |
|  | CON | -1.25 | 1.316067371 | 0 | 0 | 0 | 0 | 0 |
| Brigitta et.al 2019 | Exergame | 14.4 | 4.095700377 | 7.2 | 12 | 1296 | 1250 | 46 |
|  | CON | 5.1 | 4.93132839 | 0 | 0 | 0 | 0 | 0 |
| Nilofar et.al 2019 | Yoga | 18.6 | 7.201260751 | 2.8 | 8 | 504 | 500 | 4 |
|  | CON | 15.41 | 4.487835461 | 0 | 0 | 0 | 0 | 0 |
| Sara et.al 2019 | RT | 0.29 | 2.656739811 | 6 | 16 | 720 | 750 | -30 |
|  | AE | -2.95 | 2.42794235 | 4 | 16 | 480 | 500 | -20 |
|  | CON | -9.32 | 2.674355935 | 0 | 0 | 0 | 0 | 0 |
| Jesper et.al 2019 | Walking | 7.1 | 2.852791763 | 4.8 | 12 | 720 | 750 | -30 |
|  | CON | 1.1 | 3.656022349 | 0 | 0 | 0 | 0 | 0 |
| Cešeiko et.al 2019 | RT | 9 | 2.885275446 | 5 | 12 | 600 | 500 | 100 |
|  | CON | -2.8 | 2.962805108 | 0 | 0 | 0 | 0 | 0 |
| Morten et.al 2020 | MT | 4.7 | 1.469972152 | 4 | 12 | 720 | 750 | -30 |
|  | CON | 1.3 | 1.592720695 | 0 | 0 | 0 | 0 | 0 |
| Hong et.al 2020 | RT | 3.5 | 2.13379592 | 3.5 | 12 | 420 | 500 | -80 |
|  | CON | -3.4 | 2.198650154 | 0 | 0 | 0 | 0 | 0 |
| Elise et.al 2020 | Walking | 0 | 15.3191875 | 4.8 | 8 | 432 | 500 | -68 |
|  | CON | -3.7 | 10.8548375 | 0 | 0 | 0 | 0 | 0 |
| Abbas et.al 2020 | MT | -0.71 | 1.99752056 | 4 | 12 | 540 | 500 | 40 |
|  | CON | -1.39 | 1.771320167 | 0 | 0 | 0 | 0 | 0 |
| Lisa et.al 2021 | AE | 13.9 | 4.366157717 | 4 | 9 | 360 | 250 | 110 |
|  | CON | 8.8 | 5.483446018 | 0 | 0 | 0 | 0 | 0 |
| Lin et.al 2021 | MT | 1.25 | 0.345137651 | 3 | 8 | 405 | 500 | -95 |
|  | CON | -0.12 | 0.254764594 | 0 | 0 | 0 | 0 | 0 |
| Roxanne et.al 2021 | MT | 1.2 | 1.885112155 | 4 | 12 | 480 | 500 | -20 |
|  | CON | 0.4 | 1.436533819 | 0 | 0 | 0 | 0 | 0 |
| Sibel et.al 2021 | Yoga | 11.7 | 4.893737542 | 2.5 | 10 | 300 | 250 | 50 |
|  | CON | 3.1 | 4.6629525 | 0 | 0 | 0 | 0 | 0 |
| Denise et.al 2021 | AE | 2.12 | 3.802797696 | 4.8 | 12 | 576 | 500 | 76 |
|  | Taichi | 3 | 4.016018 | 3 | 12 | 360 | 250 | 110 |
|  | CON | -3.1 | 3.791187154 | 0 | 0 | 0 | 0 | 0 |
| Marta et.al 2022 | MT | 12 | 2.383830333 | 3.7 | 12 | 444 | 500 | -56 |
|  | CON | -0.1 | 3.809063703 | 0 | 0 | 0 | 0 | 0 |
| Dejan et.al 2022 | AE | 8 | 4.857196431 | 3.5 | 12 | 420 | 500 | -80 |
|  | CON | -3 | 4.543746619 | 0 | 0 | 0 | 0 | 0 |
| Dharam et.al 2022 | Yoga | 1.9 | 2.699338543 | 2.5 | 6 | 300 | 250 | 50 |
|  | CON | -6.3 | 3.199084244 | 0 | 0 | 0 | 0 | 0 |
| Pedro et.al 2023 | MT | 5.2 | 4.230070863 | 4 | 4 | 480 | 500 | -20 |
|  | CON | -12 | 3.762180124 | 0 | 0 | 0 | 0 | 0 |
| Joachim et.al 2016 | AE | 17.4 | 4.92386722 | 4.3 | 3 | 645 | 750 | -105 |
|  | AE | 9.5 | 3.576959162 | 3.8 | 3 | 525 | 500 | 25 |
| Holyan et.al 2017 | BME | 6.07 | 1.731048815 | 3 | 8 | 360 | 250 | 110 |
|  | RT | 4.27 | 1.598487827 | 4 | 8 | 480 | 500 | -20 |
| Alyson et.al 2007 | BME | -1.26 | 18.71981036 | 2.8 | 12 | 504 | 500 | 4 |
|  | CON | -4.46 | 21.87419987 | 0 | 0 | 0 | 0 | 0 |

# Supplementary File 4: Three key assumptions of network meta-analysis.

## Consistency

We analyzed the data to assess consistency, specifically the network effect sizes, and the network's uncorrelated mean effect (UME) model, which considers pairwise effect sizes(Higgins et al., 2012). We compared the consistency of these models by examining the deviations, the number of estimated parameters in the network, and the Deviance Information Criterion (DIC) metrics. The results indicated a good fit, as these parameters showed good agreement between the models (Supplementary Table 1). In addition to visually verifying the consistency of the model, using the residuals, a scatterplot shape was plotted (Supplementary 4 Figure 3).

### Supplementary File 4 Table 1. Consistent and UME models fit comparison.

| **Model** | **pD** | **Residual deviance** | **DIC** | **SD** |
| --- | --- | --- | --- | --- |
| **Consistent** | **85.7** | **95.674** | **558.2** | **0.405** |
| **UME** | **82.6** | **96.658** | **556.4** | **0.395** |


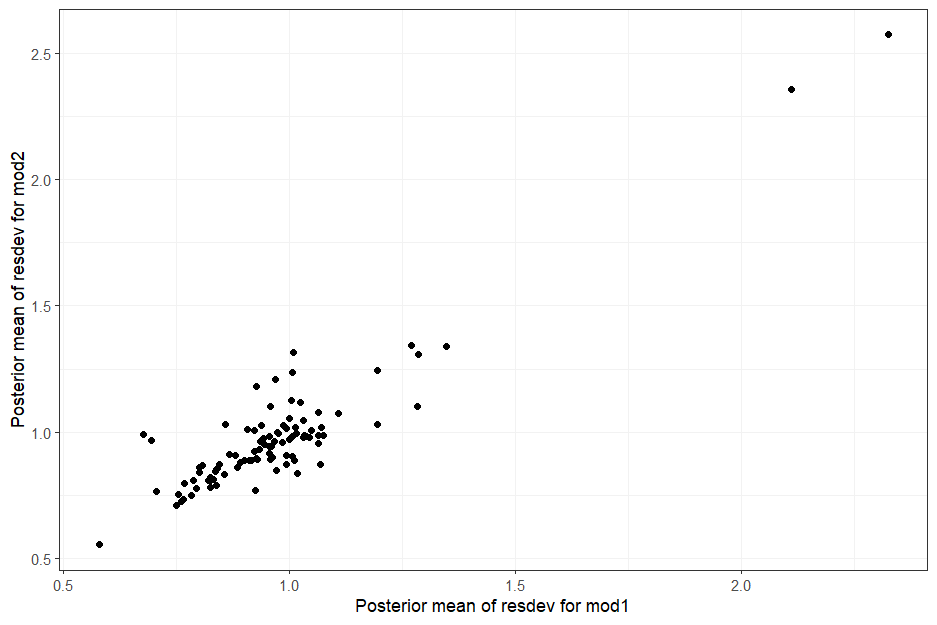


## Transitivity

Transitivity NMA is based on the assumption of potential indirect/mixed comparisons, which implies that direct and indirect evidence are consistent in their estimates of treatment effects, but with the usual variability that occurs in meta-analyses under random effects models(Shim et al., 2017). This assumption is equivalent to heterogeneity in "standard" meta-analyses. Following previous recommendations, transmissibility was assessed at a deeper level of the network (i.e., the treatment level). We assessed transitivity through the MBNMA node splitting method. This method divides and compares the contribution of a given treatment contrast into direct and indirect evidence. Similar effects indicate good transitivity. Supplementary Table 2 and Figure 4 (density plot) give the transitivity results of this meta-analysis.

### Supplementary File 4 Figure 1. Validation Model Consistency Scatterplot.

### Supplementary File 4 Table 2. Comparison of transitivity.

| Comparison |  | P value | Median | 2.50% | 97.50% |
| --- | --- | --- | --- | --- | --- |
| RT_500 vs BME_500 |  | 0.557 |  |  |  |
| > direct |  |  | 0.213 | 1.137 | 0.7 |
| > indirect |  |  | 0.126 | 0.361 | 0.6 |
| > MBNMA |  |  | 0.057 | 0.37 | 0.478 |
|  |  |  |  |  |  |
| MT_500 vs BME_250 |  | 0.463 |  |  |  |
| > direct |  |  | 0.006 | 1.064 | 1.101 |
| > indirect |  |  | 0.228 | 0.102 | 0.571 |
| > MBNMA |  |  | 0.203 | 0.122 | 0.545 |
|  |  |  |  |  |  |
| RT_500 vs AE_500 |  | 0.609 |  |  |  |
| > direct |  |  | 0.027 | 1.107 | 1.15 |
| > indirect |  |  | 0.032 | 0.422 | 0.514 |
| > MBNMA |  |  | 0.035 | 0.364 | 0.47 |
|  |  |  |  |  |  |
| AE_750 vs AE_500 |  | 0.038 |  |  |  |
| > direct |  |  | 0.568 | 1.614 | 0.465 |
| > indirect |  |  | 0.017 | 0.001 | 0.058 |
| > MBNMA |  |  | 0.016 | 0.001 | 0.057 |
|  |  |  |  |  |  |
| RT_750 vs Placebo_0 |  | 0.693 |  |  |  |
| > direct |  |  | 0.435 | 0.443 | 1.28 |
| > indirect |  |  | 0.512 | 0.095 | 0.926 |
| > MBNMA |  |  | 0.494 | 0.143 | 0.869 |
|  |  |  |  |  |  |
| RT_250 vs Placebo_0 |  | 0.613 |  |  |  |
| > direct |  |  | 0.562 | 0.134 | 1.26 |
| > indirect |  |  | 0.375 | 0.025 | 0.723 |
| > MBNMA |  |  | 0.413 | 0.118 | 0.743 |
|  |  |  |  |  |  |
| MT_1250 vs Placebo_0 |  | 0.409 |  |  |  |
| > direct |  |  | 0.431 | 0.541 | 1.401 |
| > indirect |  |  | 0.646 | 0.393 | 0.918 |
| > MBNMA |  |  | 0.632 | 0.393 | 0.888 |
|  |  |  |  |  |  |
| MT_1000 vs Placebo_0 |  | 0.4 |  |  |  |
| > direct |  |  | 0.852 | 0.017 | 1.719 |
| > indirect |  |  | 0.602 | 0.361 | 0.842 |
| > MBNMA |  |  | 0.623 | 0.389 | 0.872 |
|  |  |  |  |  |  |
| MT_750 vs Placebo_0 |  | 0.412 |  |  |  |
| > direct |  |  | 0.789 | 0.403 | 1.176 |
| > indirect |  |  | 0.507 | 0.238 | 0.798 |
| > MBNMA |  |  | 0.606 | 0.381 | 0.839 |
|  |  |  |  |  |  |
| MT_250 vs Placebo_0 |  | 0.323 |  |  |  |
| > direct |  |  | 0.178 | 0.711 | 1.101 |
| > indirect |  |  | 0.518 | 0.322 | 0.766 |
| > MBNMA |  |  | 0.498 | 0.305 | 0.72 |
|  |  |  |  |  |  |
| BME_750 vs Placebo_0 |  | 0.415 |  |  |  |
| > direct |  |  | 0.118 | 0.79 | 1.007 |
| > indirect |  |  | 0.48 | 0.186 | 0.808 |
| > MBNMA |  |  | 0.435 | 0.129 | 0.734 |
|  |  |  |  |  |  |
| AE_250 vs Placebo_0 |  | 0.2 |  |  |  |
| > direct |  |  | 1.004 | 0.133 | 1.877 |
| > indirect |  |  | 0.344 | 0.099 | 0.599 |
| > MBNMA |  |  | 0.39 | 0.151 | 0.641 |

# Supplementary File 5：Nonlinear functions and models fit comparison.


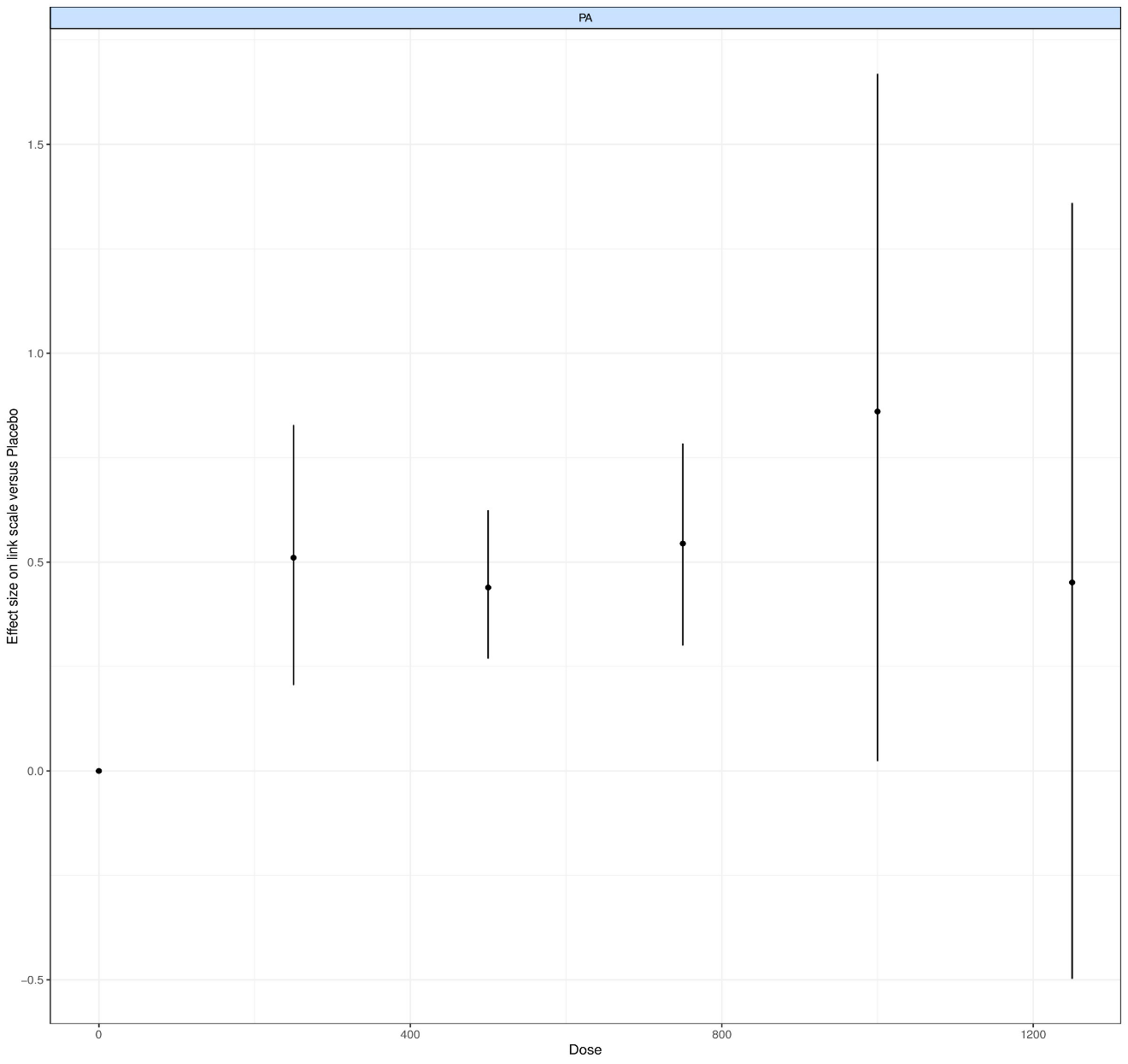
Meta-analyses (i.e., "split" NMAs) of different doses of exercise as independent and unrelated treatments were performed. This step helps to determine which function is better suited to the data and is subsequently used in a model-based network meta-analysis (MBNMA). Supplementary 5 Fig. 1 and Supplementary 5 Fig. 2 show the different responses of each dose to overall exercise and to different types of exercise.

### Supplementary File 5 Figure 1. “Split” NMA of overall exercise.


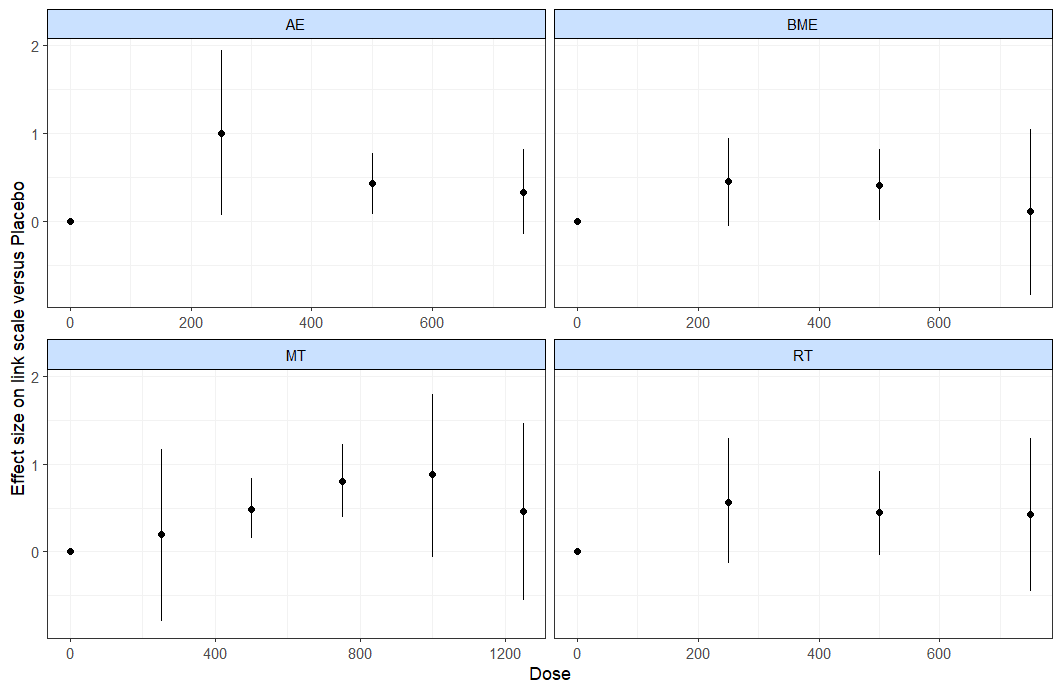


### Supplementary File 5 Figure 2. “Split” NMA of different exercises.

Table 5.1 shows the fit indices from each of the models fitted. For our data, restricted cubic splines show the best fit and were therefore used in subsequent analyses.

### Table 5.1. Models fit comparison.

| Model | DIC | SD | Deviance | Residual deviance | pD |
| --- | --- | --- | --- | --- | --- |
| Emax  (common treatment effects) | 611.9 | NA | 560.474 | 182.931 | 52.1 |
| Emax  (random treatment effects) | 550.5 | 0.364 | 471.801 | 94.258 | 79.2 |
| Exponential  (common treatment effects) | 612.4 | NA | 561.362 | 183.819 | 51.4 |
| Exponential  (random treatment effects) | 550.4 | 0.363 | 471.529 | 93.987 | 79.8 |
| Restricted cubic spline  (common treatment effects; 3 knots) | 612.1 | NA | 556.067 | 178.525 | 56.7 |
| Restricted cubic spline  (random treatment effects; 3 knots) | 549.5 | 0.377 | 472.726 | 95.183 | 82.6 |
| Non-parametric monotonically up (common treatment effects) | 614.7 | NA | 560.257 | 182.715 | 55.0 |
| Non-parametric monotonically up (random treatment effects) | 555.2 | 0.409 | 473.564 | 96.021 | 85.5 |
| Linear (common treatment effects) | 645.8 | NA | 557.344 | 341.387 | 89.1 |
| Linear (random treatment effects) | 554.5 | 0.388 | 474.170 | 96.627 | 81.0 |

DIC = Deviance Information Criterion; SD = Between-study Standard Deviation; pD: Number of estimated parameters; NA = Not Applicable. The SD is presented as the main value and (95% Credible Intervals).


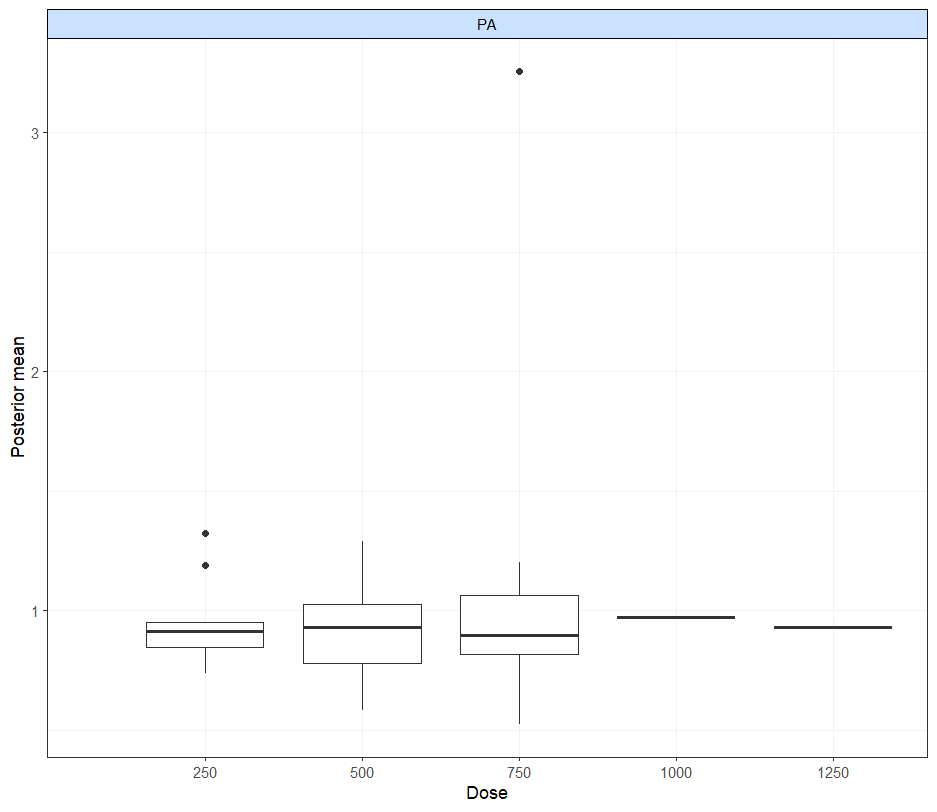
In addition to the model fit indices, the bias plots showing the contribution of each data point to the residuals also help to confirm the robustness of the model choice(Dias, Sutton, et al., 2013; Dias, Welton, et al., 2013). The contribution of each data point to the deviation from the posterior mean is approximately 1, which indicates a good model fit. Deviation plots for overall (Supplementary 5 Figure 7) and treatment effect (Supplementary 5 Figure 8) confirm the robustness of our model choice.

### Supplementary File 5 Figure 3. Deviance box plot at overall exercise level.


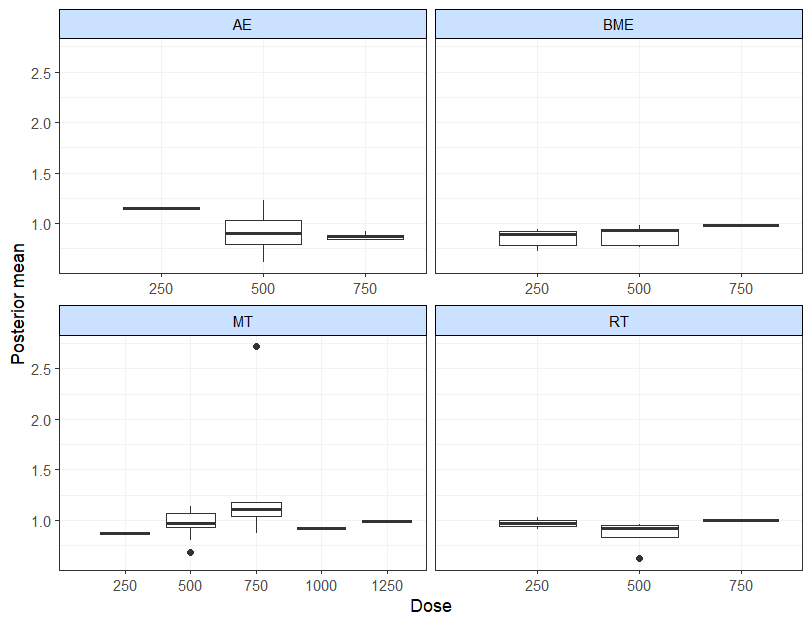


### Supplementary File 5 Figure 4. Deviance box plot at different exercise levels.

#
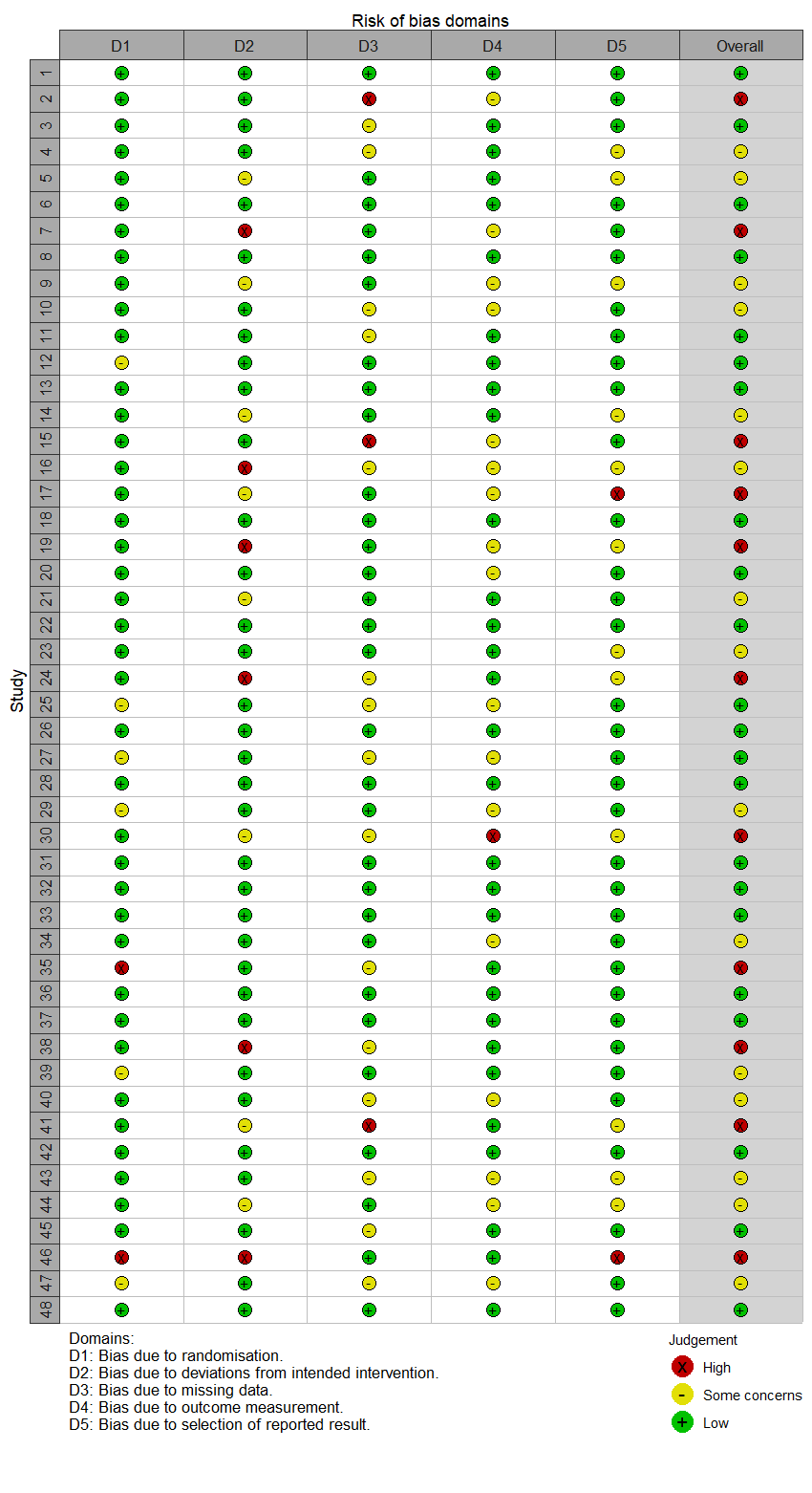
Supplementary File 6: Study level Risk of Bias analysis.

### Supplementary File 6 Figure 1: Study level Risk of Bias analysis.

# Supplementary File 7: Inconsistency and heterogeneity.

|  |  |  | NMA | | | Direct | | | Indirect | | | Diff | | | |
| --- | --- | --- | --- | --- | --- | --- | --- | --- | --- | --- | --- | --- | --- | --- | --- |
| **Comparison** | k | Prop | TE | seTE | p | TE | seTE | p | TE | seTE | p | TE | seTE | p | z |
| AE vs BME | 0 | 0 | 0.0456188 | 0.1751355 | 0.7944958 | NA | NA | NA | 0.0456188 | 0.1751355 | 0.7944958 | NA | NA | NA | NA |
| AE vs CON | 13 | 0.9526114 | 0.431372 | 0.1224934 | 0.000429 | 0.4326606 | 0.1255032 | 0.000566 | 0.4054673 | 0.5626984 | 0.4711701 | 0.0271934 | 0.5765245 | 0.9623795 | 0.0471677 |
| AE vs MT | 0 | 0 | -0.107453 | 0.1584066 | 0.4975585 | NA | NA | NA | -0.107453 | 0.1584066 | 0.4975585 | NA | NA | NA | NA |
| AE vs RT | 1 | 0.127062 | -0.019533 | 0.1920071 | 0.9189721 | -0.043271 | 0.5386535 | 0.9359738 | -0.016077 | 0.2055065 | 0.9376427 | -0.027193 | 0.5765245 | 0.9623795 | -0.047168 |
| BME vs CON | 11 | 0.8608259 | 0.3857532 | 0.1261139 | 0.0022225 | 0.3456929 | 0.1359269 | 0.0109834 | 0.6335355 | 0.3380522 | 0.0609199 | -0.287843 | 0.3643561 | 0.4295257 | -0.790004 |
| BME vs MT | 1 | 0.0928832 | -0.153072 | 0.157485 | 0.3310623 | 0.0084878 | 0.5167381 | 0.9868947 | -0.169614 | 0.1653513 | 0.3049942 | 0.1781022 | 0.542549 | 0.742708 | 0.3282694 |
| BME vs RT | 1 | 0.2146626 | -0.065151 | 0.1888798 | 0.7301427 | 0.1946373 | 0.4076687 | 0.6330489 | -0.136162 | 0.2131363 | 0.5229223 | 0.3307989 | 0.4600227 | 0.4720839 | 0.7190926 |
| MT vs CON | 16 | 0.9644336 | 0.5388249 | 0.1004835 | 8.22E-08 | 0.5451593 | 0.1023196 | 9.93E-08 | 0.3670571 | 0.5328134 | 0.4908838 | 0.1781022 | 0.542549 | 0.742708 | 0.3282694 |
| RT vs CON | 5 | 0.7875213 | 0.4509046 | 0.1561152 | 0.0038735 | 0.4967491 | 0.1759196 | 0.0047469 | 0.2809887 | 0.3386785 | 0.4067299 | 0.2157604 | 0.3816423 | 0.5718376 | 0.5653472 |
| MT vs RT | 0 | 0 | 0.0879203 | 0.18519 | 0.6349599 | NA | NA | NA | 0.0879203 | 0.18519 | 0.6349599 | NA | NA | NA | NA |

***Note.*** The SIDE method is a metric used to evaluate the performance of a classifier and is particularly effective in applications with imbalanced datasets. In Bayesian network meta-analysis, the SIDE method can also be used to detect inconsistencies. The basic principle is to segment the data into different segments or subsets, and then evaluate the errors in each subset to identify and quantify inconsistencies. Therefore, we used SIDE (Segmented Indicator for Data Error) to explore global inconsistency in depth. Comparison: Treatment comparison, k: Number of studies providing direct evidence, prop: Direct evidence proportion, NMA: Estimated treatment effect (SMD) in network meta-analysis, Direct: Estimated treatment effect (SMD) derived from direct evidence, Indirect: Estimated treatment effect (SMD) derived from indirect evidence, Diff: Difference between direct and indirect treatment estimates, Z: z-value of test for disagreement (direct versus indirect), p-value: p-value of test for disagreement (direct versus indirect).

* Within-Design Q Statistic: Q Statistics: The Q statistic is a measure of heterogeneity among studies. It tests whether the observed variation in study results is greater than expected by chance. A high Q value indicates significant heterogeneity, meaning that the results across studies are not consistent. df (Degrees of Freedom): Degrees of freedom, typically the number of studies minus one. Higher degrees of freedom indicate more independent information in the model. P-value: The p-value is the probability of observing the test results under the null hypothesis. A p-value less than a significance level (usually 0.05) indicates significant heterogeneity.

* Between-Design Q Statistic: Q Statistics: The Q statistic between designs measures heterogeneity across different study designs. Unlike the within-design Q statistic, it focuses on the differences between designs rather than within a single design. df (Degrees of Freedom): Degrees of freedom represent the number of independent comparisons minus parameters. More degrees of freedom indicate a larger number of comparisons. P-value: The p-value indicates the significance of heterogeneity between designs. A p-value less than 0.05 suggests significant differences between designs.

* Heterogeneity Measures: I^2: The I^2 statistic represents the percentage of total variation across studies due to heterogeneity rather than chance. The range is from 0% to 100%, where higher values indicate greater heterogeneity. Common thresholds are: 0%-25%: Low heterogeneity, 25%-50%: Moderate heterogeneity, 50%-75%: High heterogeneity, 75%-100%: Very high heterogeneity, tau^2: Tau-squared (τ²) is the variance of true effect sizes across studies. Higher τ² indicates greater variability between study results. tau: Tau (τ) is the standard deviation of true effect sizes. Higher τ values indicate greater dispersion of effect sizes across studies.

* Full Design-by-Treatment Interaction Random Effects Model [4]: Q Statistics: The Q statistic in this context measures consistency under the assumption of a full design-by-treatment interaction random effects model, considering all interactions between designs and treatments. df (Degrees of Freedom): Degrees of freedom for this Q statistic. P-value: The p-value tests whether the observed interaction effects are significantly different from zero. tau. within: Within-design tau (τ) represents the standard deviation of effect sizes within a single design. tau2.within: Within-design tau-squared (τ²) represents the variance of effect sizes within a single design.

| **Treatment estimate (sm = 'SMD', comparison: other treatments vs 'CON'):** | | | | |
| --- | --- | --- | --- | --- |
|  | SMD | 95% CI | z | p-value |
| AE | 0.4314 | [0.1913; 0.6715] | 3.52 | 0.0004 |
| BME | 0.3858 | [0.1386; 0.6329] | 3.06 | 0.0022 |
| CON | NA | NA | NA | NA |
| MT | 0.5388 | [0.3419; 0.7358] | 5.36 | <0.0001 |
| RT | 0.4509 | [0.1449; 0.7569] | 2.89 | 0.0039 |
| **Quantifying heterogeneity / inconsistency:** | | | | |
| tau^2 = 0.0992; | tau = 0.3149; | **I^2 = 58.9% [43.0%; 70.4%]** |  |  |
| **Tests of** **heterogeneity (within designs) and inconsistency (between designs):** | | | | |
|  | Q | d.f. | p-value |  |
| Total | 107.12 | 44 | <0.0001 |  |
| With designs | 105.91 | 41 | <0.0001 |  |
| Between designs | 1.21 | 3 | 0.7504 |  |
|  |  |  |  |  |
| **Design-specific decomposition of within-designs Q statistic:** | | | | |
|  | Q | d.f. | p-value |  |
| CON vs MT | 74.94 | 15 | <0.0001 |  |
| CON vs AE | 20.1 | 12 | 0.0653 |  |
| CON vs RT | 3.49 | 4 | 0.48 |  |
| CON vs BME | 7.39 | 10 | 0.6884 |  |
|  |  |  |  |  |
| **Between-designs Q statistic after detaching of single designs:** | | | | |
|  | Q | d.f. | p-value |  |
| BME vs RT | 0.06 | 2 | 0.9681 |  |
| CON vs BME | 0.18 | 2 | 0.9153 |  |
| CON vs RT | 0.46 | 2 | 0.7943 |  |
| BME vs MT | 1.18 | 2 | 0.5545 |  |
| CON vs MT | 1.18 | 2 | 0.5545 |  |
| CON vs AE | 1.2 | 2 | 0.5496 |  |
| AE vs RT | 1.2 | 2 | 0.5496 |  |
|  |  |  |  |  |
| **Q statistic to assess consistency  under the assumption of a full design-by-treatment interaction random effects model:** | | | | |
| Q | d.f. | p-value | tau.within | tau^2.within |
| 0.64 | 3 | 0.8867 | 0.3272 | 0.107 |

# Supplementary File 8: Exercise programs Definitions.

| **Exercise Types** | **Definitions** |
| --- | --- |
| Aerobic Exercise (AE) | AE is defined as a repetitive, structured physical activity that requires the body's metabolic system to use oxygen to produce energy (Millstein, 2020). |
| Resistance Training (RT) | RT refers to a specialized method of conditioning which involves the progressive use of a wide range of resistive loads and a variety of training modalities designed (Steele et al., 2017). |
| Multicomponent Training (MT) | MT is a training modality that involves different physical capacities in the same exercise session. For example, including AE and RT in a session (Li et al., 2023). |
| Body-Mind Exercise (BME) | BME are practices that use movements and/or postures along with a focus on breathing to achieve a meditative state. For example, Qigong, Tai Chi, and Yoga can be defined as BME (Yin et al., 2021, Xu et al., 2024). |

1: LI, G., YOU, Q., HOU, X., ZHANG, S., DU, L., LV, Y. & YU, L. 2023. The effect of exercise on cognitive function in people with multiple sclerosis: a systematic review and meta-analysis of randomized controlled trials. J Neurol, 270, 2908-2923.
2: MILLSTEIN, R. 2020. Aerobic exercise. Encyclopedia of Behavioral Medicine. Springer.
STEELE, J., FISHER, J., GIESSING, J. & GENTIL, P. 2017. Clarity in reporting terminology and definitions of set endpoints in resistance training. Muscle Nerve, 56, 368-374.
3: XU, H., LIU, J., LI, P. & LIANG, Y. 2024. Effects of mind-body exercise on perimenopausal and postmenopausal women: a systematic review and meta-analysis. Menopause, 31, 457-467.
4: YIN, Z., MARTINEZ, C. E., LI, S., MARTINEZ, M., PENG, K., LAND, W. M., ULLEVIG, S. L., CANTU, A., FALK, S., HERNÁNDEZ, A. E., ORTEGA, C., PARRA-MEDINA, D. & SIMMONDS, M. J. 2021. Adapting Chinese Qigong Mind-Body Exercise for Healthy Aging in Older Community-Dwelling Low-income Latino Adults: Pilot Feasibility Study. JMIR Aging, 4, e29188.

Supplementary File 9: Sensitivity analysis.

#
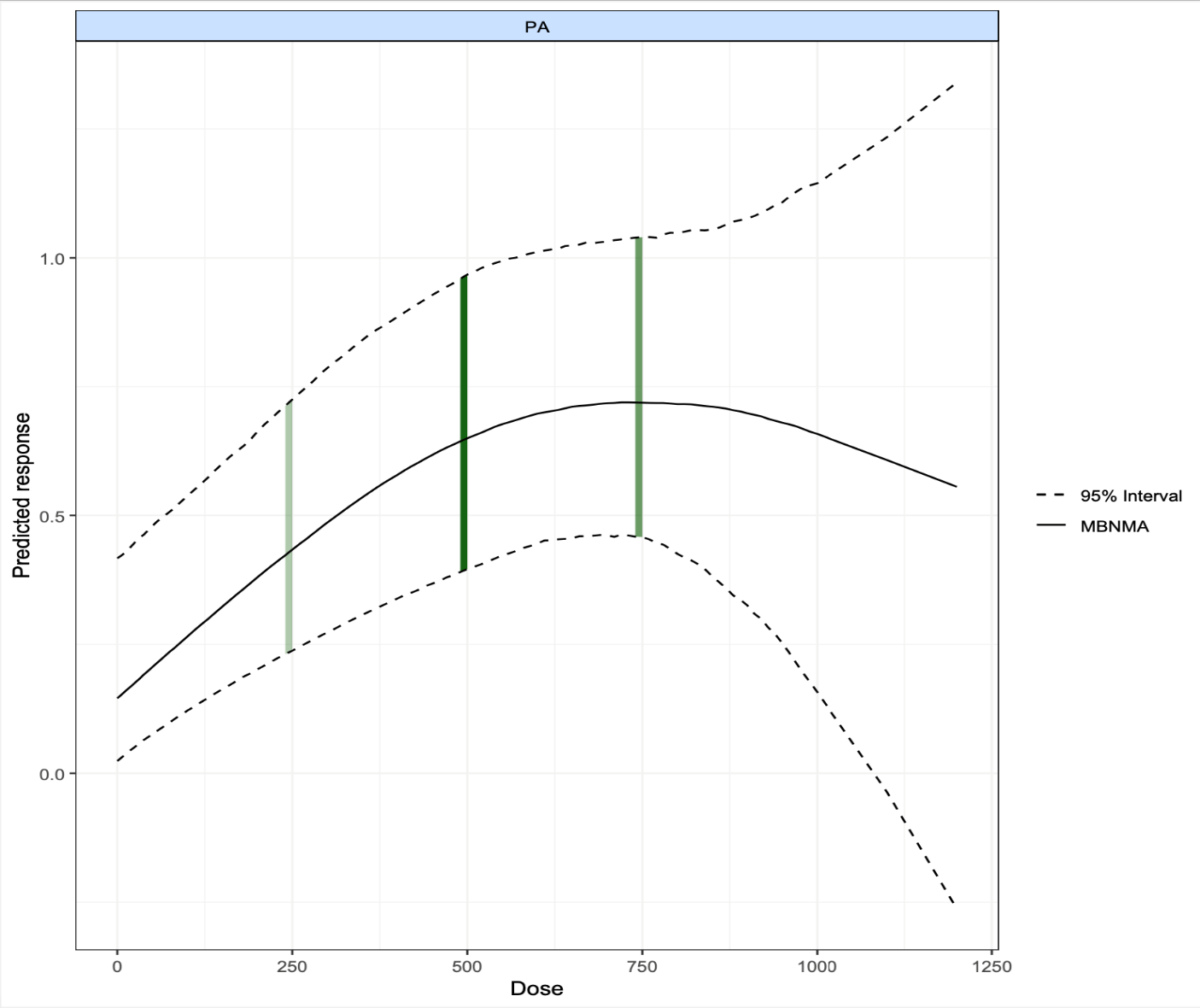


# Supplementary File 9 Figure 1. Sensitivity analysis of overall exercise dose-response relationship curve.

#
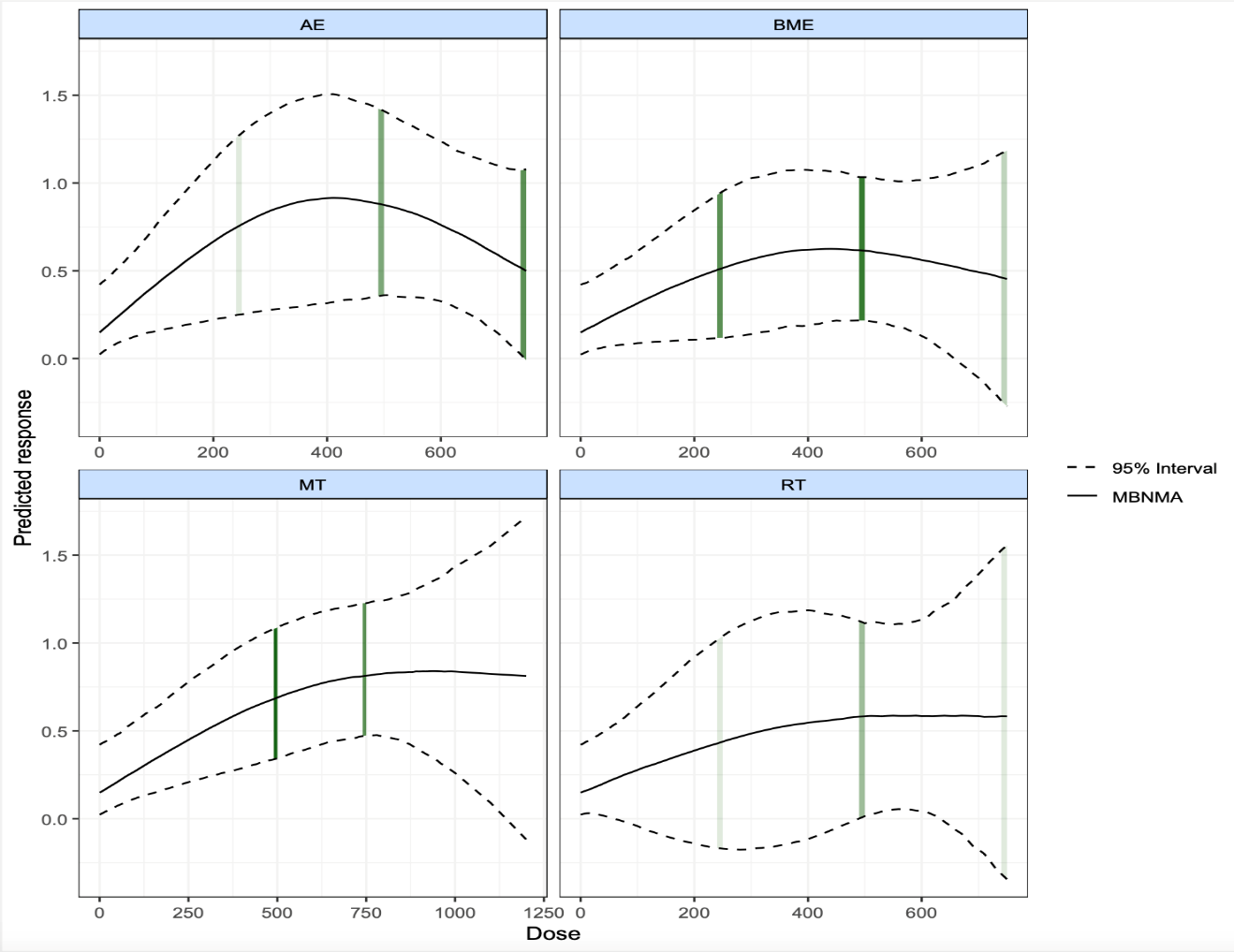
Supplementary File 9 Figure 2. Sensitivity analysis of different exercise dose-response relationship curves.

# Reference

Dias, S., Sutton, A. J., Ades, A. E., & Welton, N. J. (2013). Evidence synthesis for decision making 2: a generalized linear modeling framework for pairwise and network meta-analysis of randomized controlled trials. *Med Decis Making*, *33*(5), 607-617. <https://doi.org/10.1177/0272989x12458724>

Dias, S., Welton, N. J., Sutton, A. J., & Ades, A. E. (2013). Evidence synthesis for decision making 1: introduction. *Med Decis Making*, *33*(5), 597-606. <https://doi.org/10.1177/0272989x13487604>

Higgins, J. P., Jackson, D., Barrett, J. K., Lu, G., Ades, A. E., & White, I. R. (2012). Consistency and inconsistency in network meta-analysis: concepts and models for multi-arm studies. *Res Synth Methods*, *3*(2), 98-110. <https://doi.org/10.1002/jrsm.1044>

Rouse, B., Chaimani, A., & Li, T. (2017). Network meta-analysis: an introduction for clinicians. *Intern Emerg Med*, *12*(1), 103-111. <https://doi.org/10.1007/s11739-016-1583-7>

Shim, S., Yoon, B. H., Shin, I. S., & Bae, J. M. (2017). Network meta-analysis: application and practice
using Stata. *Epidemiol Health*, *39*, e2017047. <https://doi.org/10.4178/epih.e2017047>
